# Insulin mitigates acute ischemia–induced atrial fibrillation and sinoatrial node dysfunction ex vivo

**DOI:** 10.1172/jci.insight.185961

**Published:** 2024-11-14

**Authors:** Huiliang Qiu, Fan Li, Hannah Prachyl, Alejandra Patino-Guerrero, Michael Rubart, Wuqiang Zhu

**Affiliations:** 1 Departments of Cardiovascular Medicine and Physiology and Biomedical Engineering and Center for Regenerative Biotherapeutics, Mayo Clinic Arizona, Scottsdale, Arizona, USA.; 2 Department of Pediatrics, Wells Center for Pediatric Research, Indiana University School of Medicine, Indianapolis, Indiana, USA.

**Keywords:** Cardiology, Therapeutics, Arrhythmias, Cardiovascular disease, Insulin

## Abstract

Acute atrial ischemia is a well-known cause of postoperative atrial fibrillation (POAF). However, mechanisms through which ischemia contributes to the development of POAF are not well understood. In this study, ex vivo Langendorff perfusion was used to induce acute ischemia/reperfusion in the heart to mimic POAF. Inducibility of atrial fibrillation (AF) was evaluated using programmed electrical stimulation and verified with open-atrium optical mapping. Compared with the control group without ischemia, 25 minutes of ischemia substantially increased the incidence of AF. The right atrium was more susceptible to AF than the left atrium. Administering insulin for 30 minutes before ischemia and during reperfusion with 25 minutes of ischemia greatly reduced the vulnerability to AF. However, insulin treatment during reperfusion only did not show substantial benefits against AF. Optical mapping studies showed that insulin mitigated ischemia-induced abnormal electrophysiology, including shortened action potential duration and effective refractory period, slowed conduction velocity, increased conduction heterogeneity, and altered calcium transients. In conclusion, insulin reduced the risk of acute ischemia/reperfusion–induced AF via improving the electrophysiology and calcium handling of atrial cardiomyocytes, which provides a potential therapy for POAF.

## Abstract 2

This study provided a potential approach to prevent abnormal heart beating.

## Introduction

Postoperative atrial fibrillation (POAF) is the new onset of atrial fibrillation (AF) that occurs shortly (i.e., a few minutes or days) after cardiac surgical procedures and noncardiac thoracotomy (1, 2). Approximately 20%–30% of cases of POAF occur in patients who have had cardiac surgery or coronary procedures (1, 3–5). Many cardiac procedures are associated with myocardial ischemia, and it is postulated that atrial ischemia may contribute to the development of AF (6–8). For example, AF is a complication in up to 21% of patients with ST elevation myocardial infarction, particularly in cases involving proximal occlusion in the right coronary artery affecting the atrial branches (9–11). In addition, AF was observed in patients within 24 hours after acute atrial ischemia caused by a transient occlusion of an atrial coronary artery branch during primary percutaneous coronary intervention (11). Furthermore, severe stenosis of an atrial branch artery has been found to increase the risk of AF by more than 50% in the year following diagnosis (12). Acute atrial ischemia is common during orthotopic heart transplantation (13). AF is a common type of arrhythmia after orthotopic heart transplantation (14). Together, these clinical data highlight the potential contribution of acute atrial ischemia to the development of POAF. POAF is a critical clinical issue, which is associated with an 8-fold increase in the risk of developing recurrent AF later on, a 3- to 4-fold increase in the risk of stroke, and a 3-fold higher risk of all-cause mortality (4, 5, 15–17). Current treatment approaches for POAF include control of heart rhythm or rate and anticoagulation (18, 19). However, a well-established prevention strategy for POAF is still lacking.

Insulin, a crucial component of the glucose-insulin-potassium (GIK) metabolic cocktail, has been extensively studied for its role in perioperative myocardial protection. It is well known for its ability to stabilize membrane potential and prevent arrhythmias since Sodi-Pallares et al. first proposed this idea in 1962 (20). The concept saw a resurgence in the 1990s through the ECLA Pilot and Pol-GIK trials (21). Studies have shown that insulin’s cardioprotective mechanisms include enhancing glycolysis, reducing oxidative stress, antiapoptotic effects, and antiinflammatory actions, achieved through the activation of glucose transporter 4, PI3K, ERK 1/2, and Cbl-associated protein (21–23). Jonassen et al. found that insulin reduced infarct size most effectively when it was administered during early reperfusion rather than before ischemia or during late reperfusion (22).

Intensive insulin therapy for patients undergoing cardiac surgery with diabetes or perioperative hyperglycemia resulted in a 48% reduction in mortality and a 24% decrease in POAF incidence, suggesting that insulin may reduce susceptibility to POAF (24). However, there are no data indicating whether insulin provides protection against acute atrial ischemia or ischemia-induced POAF. Based on the information provided, we hypothesize that insulin prevents acute atrial ischemia–induced AF. The objective of current study is to investigate whether and how acute atrial ischemia leads to POAF and the potential protective role of insulin in acute atrial ischemia–induced POAF. Our study was conducted in an isolated heart perfusion setting to simulate the conditions of acute atrial ischemia, followed by open-atria optical mapping to explore the mechanisms of AF.

## Results

### Acute ischemia/reperfusion increases AF susceptibility.

**Figure 1:**
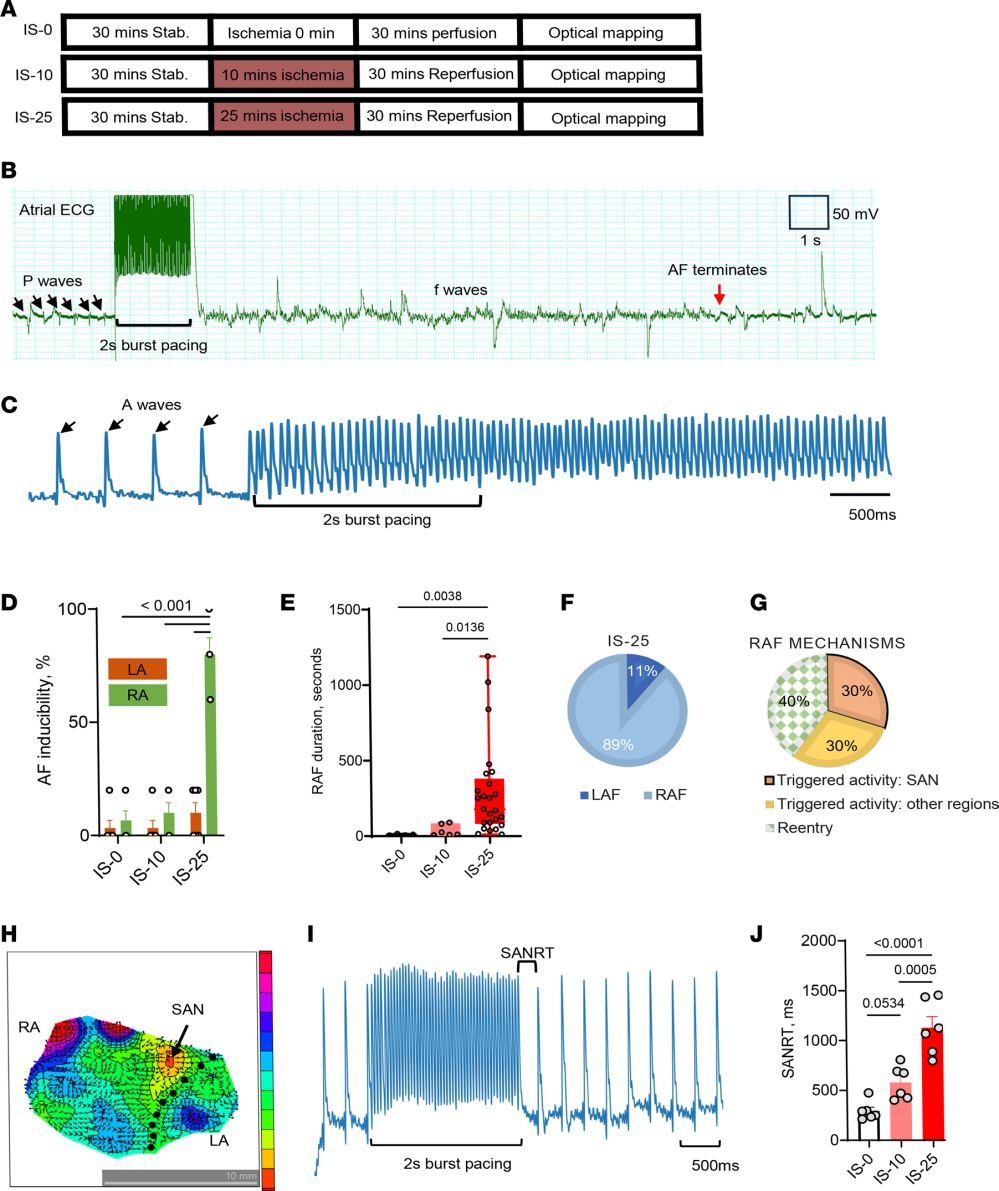
Acute atrial I/R–induced AF and SAN dysfunction. (**A**) Experimental protocol. Stab, stabilization; IS-0, no ischemia without insulin; IS-10, ischemia for 10 minutes without insulin; IS-25, ischemia for 25 minutes without insulin. (**B**) Atrial ECG of a short episode of AF induced by a 2-second 50 Hz burst pacing. (**C**) Optical mapping of action potential of an episode of AF. (**D**) AF inducibility. (**E**) RAF duration. Ischemia for 25 minutes significantly increased the susceptibility to RAF and cumulative duration of RAF. (**F**) The right atrium is 8 times more susceptible to AF than the left atrium after 25 minutes of ischemia. (**G**) We found 60% RAF episodes were due to triggered activities, half of which originated from and/or near the SAN area. (**H**) Activation map of atria; arrow indicates the identification of SAN. (**I**) Action potential of SAN (near the SAN area) and sinoatrial node recovery time (SANRT). (**J**) Impact of different ischemia duration on SANRT. Data in all panels except for **E** were presented as mean ± SEM; *n* = 6 (biological repeats) each group; statistical analysis was performed by 2-way (for **D**) or 1-way (for **J**) ANOVA, respectively, with Tukey’s multiple-comparison test. Data in **E** were presented as median [25th to 75th percentile] and analyzed by Kruskal-Wallis test; *n* = 5, 8, 25 (technical repeats) in IS-0, IS-10, and IS-25, respectively.

The main purpose of this study was to model the ischemic process of the donor heart and to determine the effect of different durations of ischemia followed by reperfusion on the susceptibility to AF. Langendorff perfusion of isolated rat hearts was used followed by endocardial optical mapping of isolated atrial preparations, allowing us to investigate the effects of ischemia/reperfusion (I/R) injury ex vivo on AF susceptibility (Supplemental Figure 1; supplemental material available online with this article; https://doi.org/10.1172/jci.insight.185961DS1). In brief, after stabilizing the baseline and administering pretreatment, global no-flow ischemia was induced for 0 minute (nonischemia, IS-0), 10 minutes (IS-10), or 25 minutes (IS-25), followed by 30 minutes of reperfusion (Figure 1A). The voltage-sensitive dye RH237 was utilized to observe changes in action potentials. The atria were then dissected, transferred to a perfusion chamber with the endocardial surface up, and continuously superfused with Tyrode’s solution. Programmed electrical stimulation was performed to assess tissue electrical conduction characteristics. Burst pacing was repeated 4 times (*n* = 5) to determine AF inducibility. An episode of flutter or fibrillation lasting over 2 seconds was considered a positive outcome. Subsequent bursts were delivered 1 minute after an AF episode terminated or 1 minute after a noninducing burst.

Representative AF episodes recorded by electrocardiography (ECG) and optical mapping are shown in Figure 1, B and C, respectively. The inducibility of AF via right atrial fibrillation (RAF) bursts among atria from hearts subjected to 25 minutes of ischemia was significantly higher than among atria subjected to 10 minutes of ischemia or no ischemia (71.67% ± 9.80% vs. 10.0% ± 3.65% in IS-10, or 3.33% ± 2.11% in IS-0, both *P* < 0.01, Figure 1D). There was no significant difference in RAF inducibility between nonischemic atria and atria undergoing 10 minutes of ischemia, and there was no significant difference in AF inducibility via left atrial burst stimulation (left atrial fibrillation, LAF) among all groups (Figure 1D). Following 25 minutes of ischemia, AF inducibility was markedly lower in the left versus right atria (8.33% ± 3.07% vs. 71.67% ± 9.80%, *P* = 0.0024, Figure 1D). Supplemental Table 1 shows occurrence of AF episodes in each animal. Although we tested the inducibility of RAF and LAF separately and AF induced from either site always propagated to the opposite site, we only counted the occurrence of AF at the atria with pacing. These data suggest that the right atrium is more vulnerable to atrial I/R–induced AF than the left atrium.

AF is considered positive when its duration lasts longer than 2 seconds to avoid false-positive results. The duration of AF was compared to evaluate the severity of AF. Ischemia for 25 minutes significantly increased the cumulative duration of RAF (178 [81 to 380.5] seconds; median [25% to 75% interquartile range]), compared with 10 minutes of ischemia (17.5 [8.25 to 84] seconds; *P* = 0.0136) or no ischemia (8 [5.25 to 13.75] seconds; *P* = 0.0038; Figure 1E). We found that the right atrium was 8 times more vulnerable than the left atrium following 25 minutes of ischemia (Figure 1F). Among the RAF episodes, 60% were identified as triggered activity–related AF. Representative images of triggered activities and reentrant AF are shown in Supplemental Figures 2 and 3. Of these, half originated from the anatomic region delineated by the sinoatrial node (SAN), superior vena cavae, and the coronary sinus (Figure 1G).

The SAN region was verified at baseline before burst pacing, and the sinoatrial node recovery time (SANRT) was collected (Figure 1, H and I). After 25 minutes of ischemia, there was a significant increase in SANRT compared with nonischemic conditions (1,129 ± 111.5 ms vs. 293.4 ± 38.7 ms, *P* = 5.39 × 10^–6^) and after 10 minutes of ischemia (1,129 ± 111.5 ms vs. 578.9 ± 68.51 ms, *P* = 0.0005) (Figure 1, I and J). Compared with nonischemic conditions, there was prolongation of SANRT after 10 minutes of ischemia, but the change did not reach statistical significance (*P* = 0.0534). These findings suggest that acute atrial I/R–induced AF adversely affects the SAN function and that ischemia duration plays a crucial role for determining AF susceptibility.

Reentrant circuits were observed in approximately 40% of cases. An illustrative activation mapping of an episode can be found in Supplemental Figure 3, A and B. This particular episode of AF was identified as a macro reentrant circuit initially resembling fast atrial arrhythmia but later transitioning into fibrillation as the action potential patterns became more erratic (Supplemental Figure 3, C and D). As depicted in Supplemental Figure 4, A–C, though ECG from the entire atria shows the episode of AF (Supplemental Figure 4A) is chaotic, the action potential of a single pixel is similar (Supplemental Figure 4B). However, the mapping revealed that each action potential followed a distinct conduction pathway (Supplemental Figure 4C), ruling out AF with a fixed origin and suggesting a combination of triggered activity, reentrant circuits, and heterogeneous tissue properties. This highlights the complexity of the mechanism of acute atrial I/R–induced reentrant AF, underscoring the substantial impact of acute atrial I/R–induced tissue damage on increasing electrical conduction variability.

### Insulin prevents I/R-induced AF.

**Figure 2:**
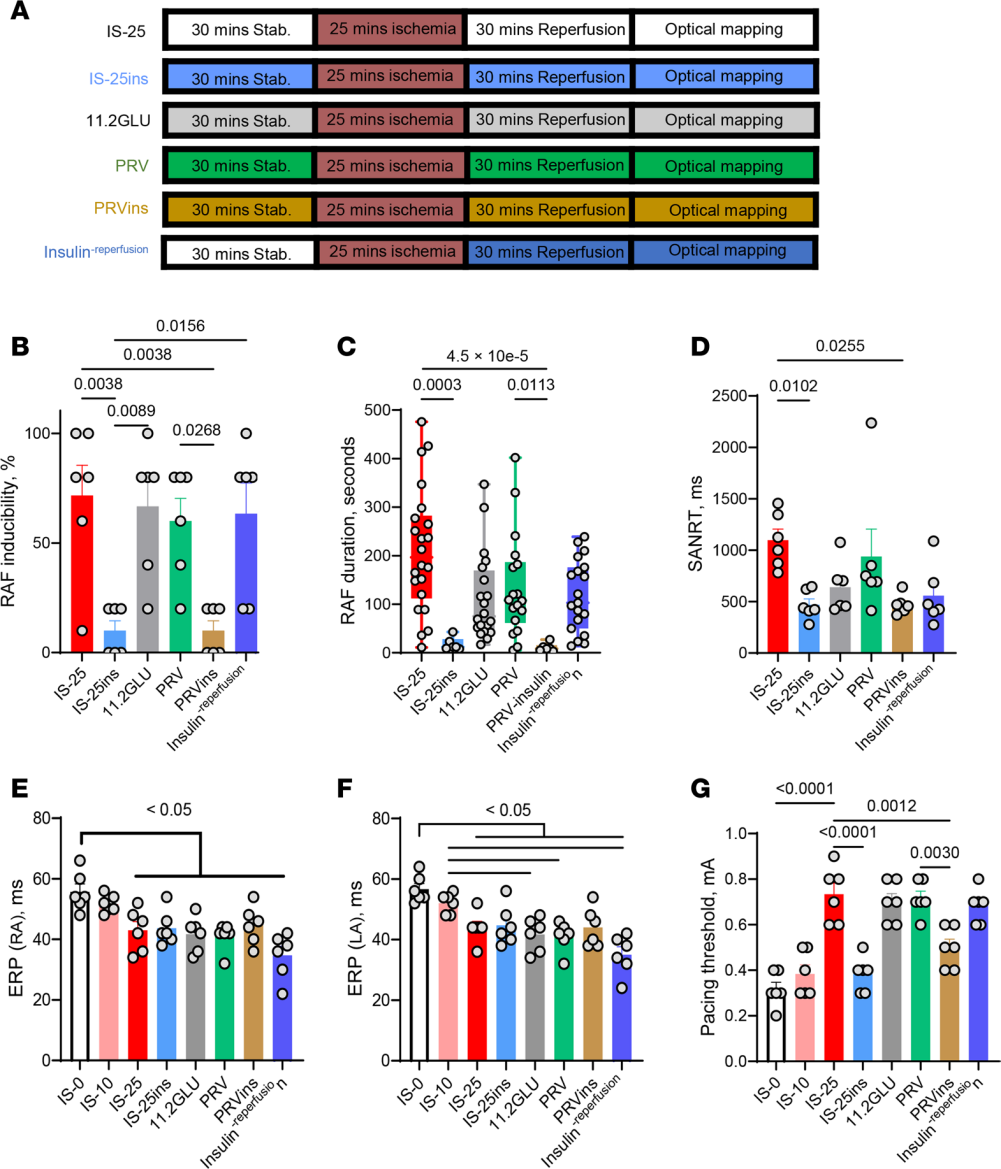
Insulin prevents I/R-induced RAF. (**A**) Experimental groups. IS-25, ischemia for 25 minutes without insulin; IS-25ins, ischemia for 25 minutes with insulin; 11.3GLU, treatment with high concentration of glucose (11.2 mM) without insulin; PRV, treatment with pyruvate without insulin; PRVins, treatment with pyruvate plus insulin; Insulin^-reperfusion^, insulin treatment during reperfusion only. (**B**) Inducibility of RAF. (**C**) RAF duration. (**D**) SANRT. (**E** and **F**) Effective refractory period (ERP). Horizontal bars represent individual comparisons (*P* < 0.05). (**G**) Pacing threshold. Data in all panels except for **C** were presented as mean ± SEM; *n* = 6 (biological repeats) per group; statistical analysis was performed by 1-way ANOVA with Tukey’s test. Data in **C** were presented as median [25 to 75 percentile]. *n* = 14, 11, 14, 6, 7, and 7 (technical repeats), respectively, in IS-25, IS-25ins, 11.2GLU, PRV, PRVins, and Insulin^-reperfusion^; statistical analysis was performed by Kruskal-Wallis test with Dunn’s multiple-comparison test.

We investigated whether insulin modulates AF inducibility by I/R. The ex vivo study enabled us to test this hypothesis using a higher insulin dose. Figure 2, A and B, show that insulin (5 mU/mL) significantly reduced RAF inducibility compared with nontreated control (10% ± 4.47% vs. 71.67% ± 13.76%, *P* = 0.0038) and shortened RAF duration (196.5 [112.3 to 282.3] seconds vs. 12 [8.75 to 28] seconds, *P* = 0.0003, Figure 2C). Supplemental Table 2 summarizes the occurrence of AF episodes in each animal. To our knowledge, this is the first report of insulin providing protection against acute atrial I/R–induced AF.

Given that insulin can directly facilitate the transport of glucose into cardiomyocytes via glucose transporters, leading to increased glucose uptake, we developed a subhypothesis that the protective effect is dependent on increased glucose uptake. Therefore, we compared the effects of higher extracellular glucose levels, replacing glucose with equimolar pyruvate, and using 2-deoxy-d-glucose (2DG; an inhibitor of glucose metabolism, data not shown), as shown in Figure 2A. Elevated glucose did not reduce susceptibility to AF compared with regular glucose (*P* = 0.9994, Figure 2B). Substituting glucose with pyruvate (5.6 mM/L) in the presence or absence of insulin showed that insulin with pyruvate prevented the onset of AF compared with insulin alone (*P* = 0.0268), suggesting that insulin’s protection against AF is not dependent on glycolysis. However, using insulin with 2DG did not show a substantial benefit, likely due to cell energy deprivation and resulting dysfunction, since glycolysis was the primary energy source in this experimental setup. Insulin with glucose or pyruvate prevented the prolongation of SANRT (Figure 2D). There were no significant differences in the effect on the refractory period among conditions with 25 minutes of ischemia (Figure 2, E and F). With insulin present, the pacing thresholds were lower than in the 25-minute ischemia condition and similar to the 10-minute ischemia condition, but this effect was not observed when insulin was administered only during reperfusion (Figure 2G). These findings suggest that the protective effect of insulin against AF is not reliant on extracellular glucose and is effective only when administered prior to ischemia.

### Insulin prevented I/R-induced electrical heterogeneity.

**Figure 3:**
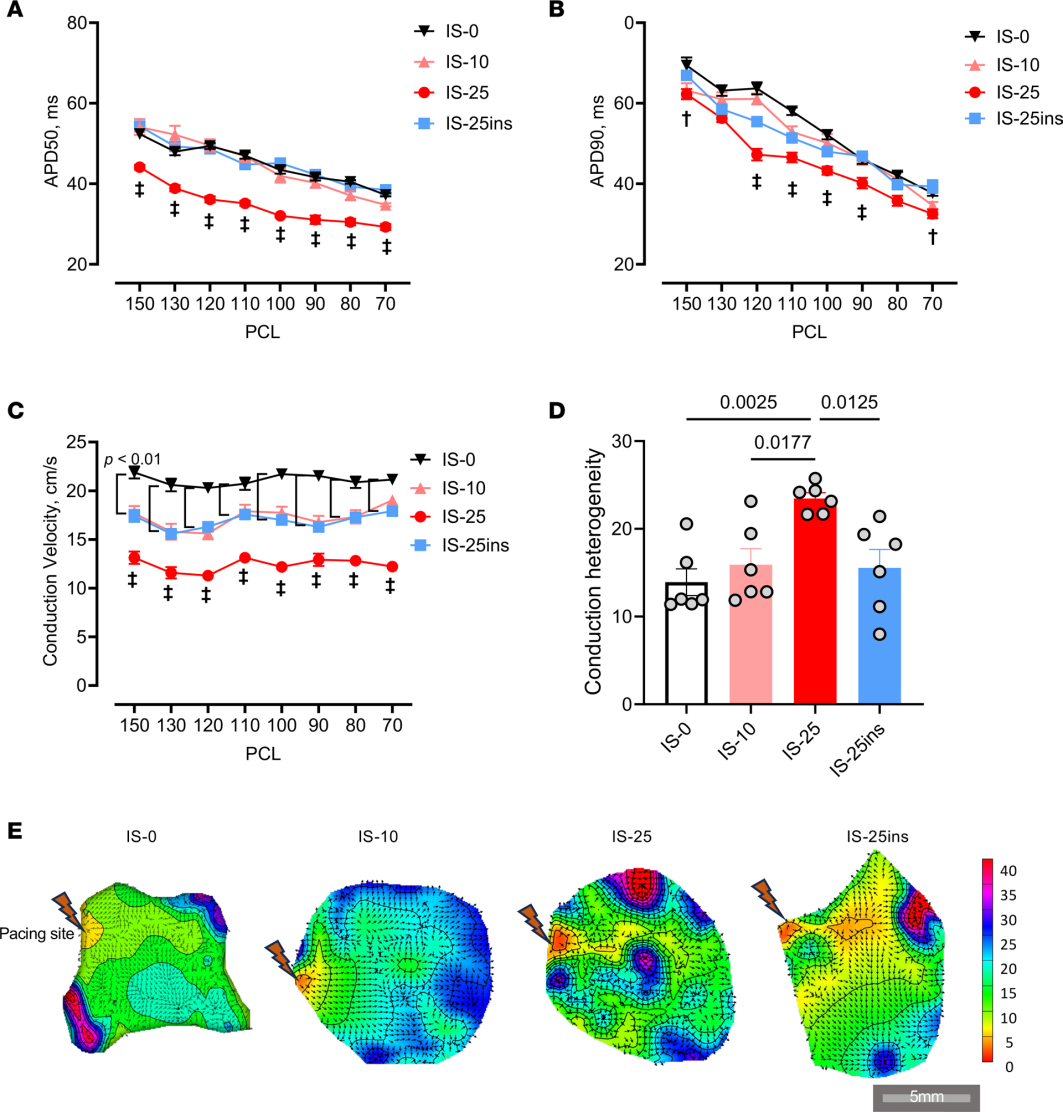
Insulin attenuates I/R-induced atrial electrical remodeling. (**A** and **B**) APD at 50% or 90% repolarization (APD50 or APD90). PCL, pacing cycling length. (**C**) Conduction velocity. (**D** and **E**) Conduction heterogeneity (**D**) and activation map (**E**) of the right atrial tissue at 130 ms PCL. Data in all panels were presented as mean ± SEM; *n* = 6 (biological repeats) each group; statistical analysis was performed by 2-way ANOVA (for **A**–**C**) or 1-way ANOVA (for **D**) with Tukey’s multiple-comparison test. ^†^*P* < 0.05, ^‡^*P* < 0.01 compared with IS-25ins.

**Figure 4:**
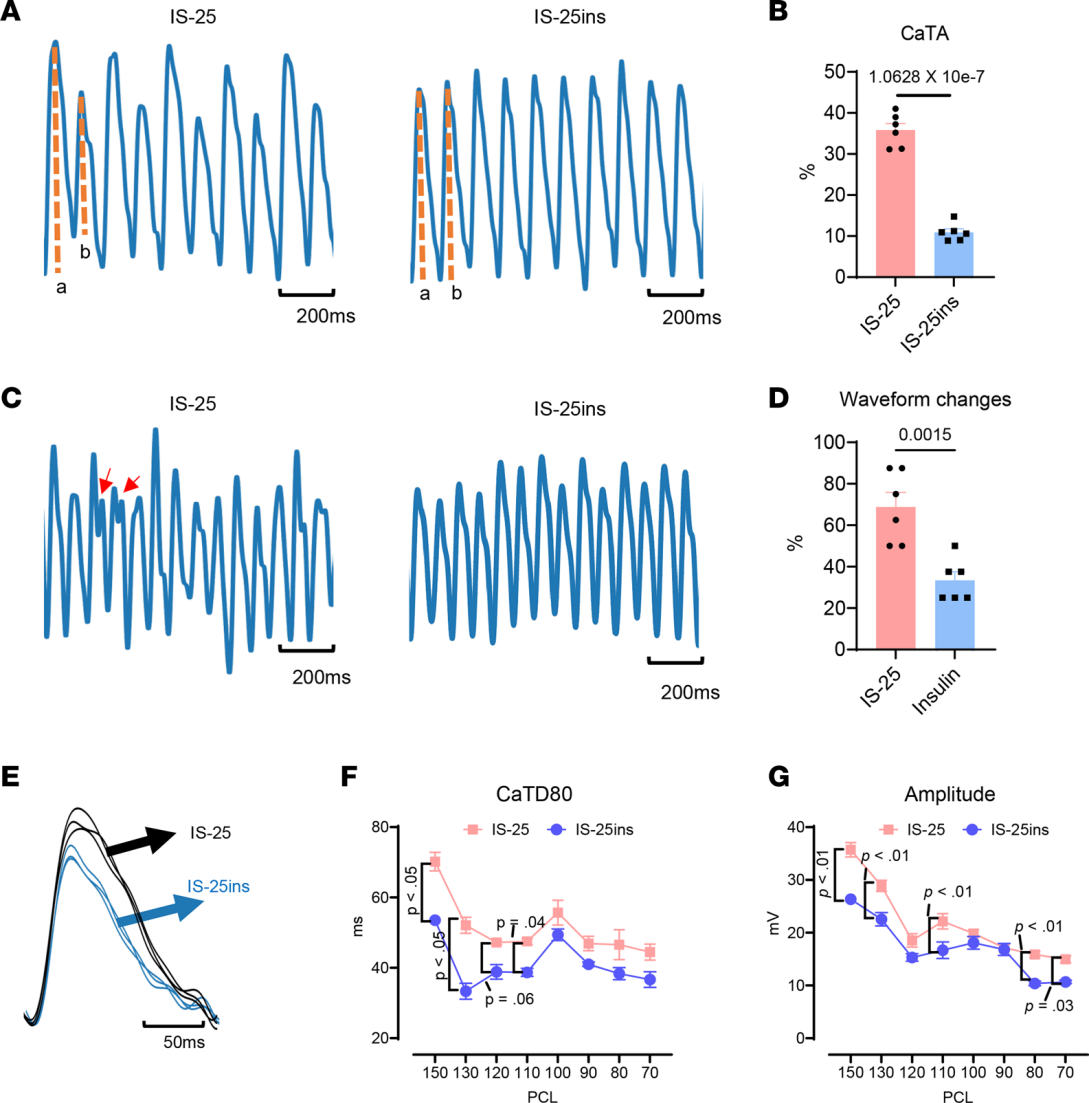
Insulin improves calcium transient in ischemic atrial tissues. (**A**–**D**) Representative recordings of calcium transient alterans (CaTA) (**A**) and waveform changes (**C**) paced at a cycling length of 130 ms and the quantification of CaTA (**B**) and waveform changes (**D**). The arrows indicate abnormal calcium transition. The percentage in **B** represents the percentage change of the later (dashed line b) from the earlier (dashed line a) calcium transient amplitude. The percentage in **D** represents the percentage waveform changes after the S1S1 pacing. (**E**) Typical calcium transients. (**F** and **G**) Calcium transient duration at 80% recovery (CaTD80) and amplitude. Data were presented as mean ± SEM; *n* = 6 (biological repeats) each group. Statistical analysis was performed by 2-tailed Student’s *t* test (for **B** and **D**) or 2-way ANOVA (for **F** and **G**) with Tukey’s multiple-comparison test.

S1S1 pacing at various cycle lengths including 150, 130, 120, 110, 100, 90, 80, and 70 ms was used to assess action potential duration (APD) and conduction velocity. As depicted in Figure 3, A–E, optical mapping demonstrated that after 25 minutes of ischemia, there was a decrease in APD at 50% and 90% repolarization (APD50 and APD90), a reduction in conduction velocity at different pacing cycle lengths (PCLs), and an increase in conduction heterogeneity. However, insulin prevented the decrease in APD and slowing of conduction velocity, resulting in improved electrical conduction homogeneity compared with the control group subjected to 25 minutes of ischemia. Separate experiments were then conducted using Rhod-2 AM dye to gather calcium transient signals. Tissues exposed to 25 minutes of ischemia without insulin showed more calcium alterations and changes in waveforms (Figure 4, A–D). Moreover, tissues exposed to 25 minutes of ischemia without insulin exhibited longer calcium transients (Figure 4, E–G).

**Figure 5:**
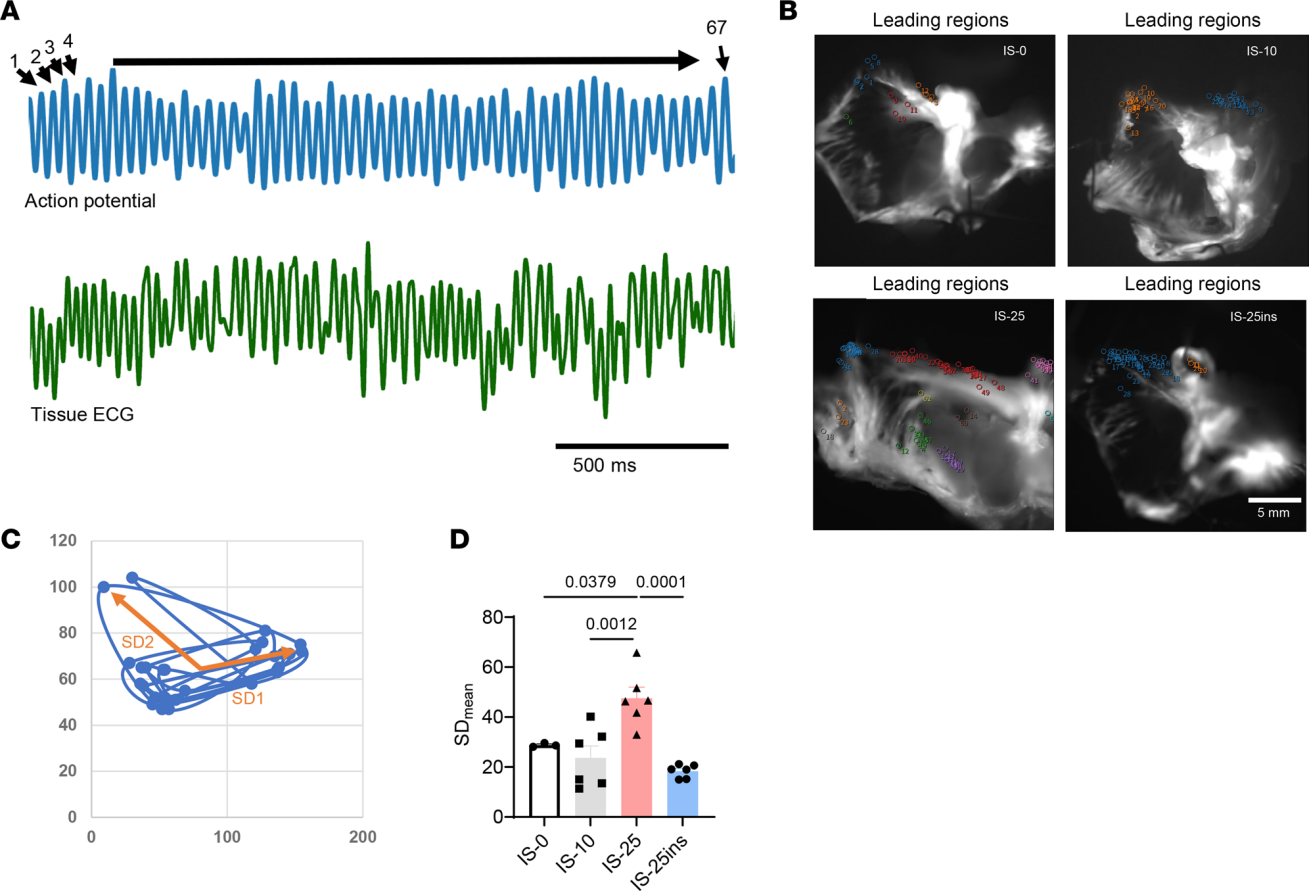
Insulin reduces the dispersion of leading regions. (**A**) Examples of AF episode’s action potentials (blue) and simultaneous ECG recording (green). (**B**) Typical leading region mappings of different conditions. (**C**) Poincaré plot of the leading regions. (**D**) Dispersion with the use of SD_mean_ value of SD1 and SD2 of the Poincaré plot. Data were presented as mean ± SEM; *n* = 3, 6, 6, and 6 (biological repeats), respectively, in IS-0, IS-10, IS-25, and IS-25ins; statistical analysis was performed by 1-way ANOVA with Tukey’s multiple-comparison test.

We analyzed the primary regions of an episode of AF. Primary regions are the areas of the heart that are the sources or focal points of an arrhythmic event, as illustrated in Figure 5, A and B. To compare the distribution of the primary regions, these points were plotted for Poincaré plot analysis. The standard deviations SD1 and SD2 were calculated from these plots to provide insight into the variability of the leading regions. The mean SD (SD_mean_) was then determined to explain the distribution of the primary regions (Figure 5C). The results showed that ischemia for 25 minutes increased the SD_mean_ value compared with the nonischemic control (*P* = 0.0379, Figure 5D) or those with 10 minutes of ischemia (*P* = 0.0012). A significantly lower SD_mean_ was observed in the insulin-treated group (*P* = 0.0001). These findings quantified the distribution of the primary regions in an episode of AF. High dispersion suggests that the AF episodes originate from multiple and varied locations, while low dispersion indicates that the arrhythmic events are more localized or confined to specific areas. Therefore, these results suggest that electrical heterogeneity within the atrial tissue is severe in those under 25 minutes of ischemia, whereas insulin-treated tissue shows larger conduction homogeneity. This increased heterogeneity could contribute to the increased susceptibility and complexity of AF under prolonged ischemic conditions.

### Insulin prevents I/R-induced SAN dysfunction.

Understanding spontaneous AF during and after ischemia is crucial. To quantify the severity of spontaneous AF, an arrhythmia scoring system was developed: 0 for none, 1 for an episode lasting less than 2 seconds, 2 for greater than or equal to 2 but less than 15 seconds, 3 for greater than or equal to 15 but less than 30 seconds, 4 for greater than or equal to 30 but less than 60 seconds, 5 for greater than or equal to 60 but less than 120 seconds, and 6 for greater than or equal to 120 seconds. Additionally, the stop time of sinus rhythm (SRST) after ischemia and recovery time of sinus rhythm (SRRT) after reperfusion were measured to evaluate the function of the SAN.

**Figure 6:**
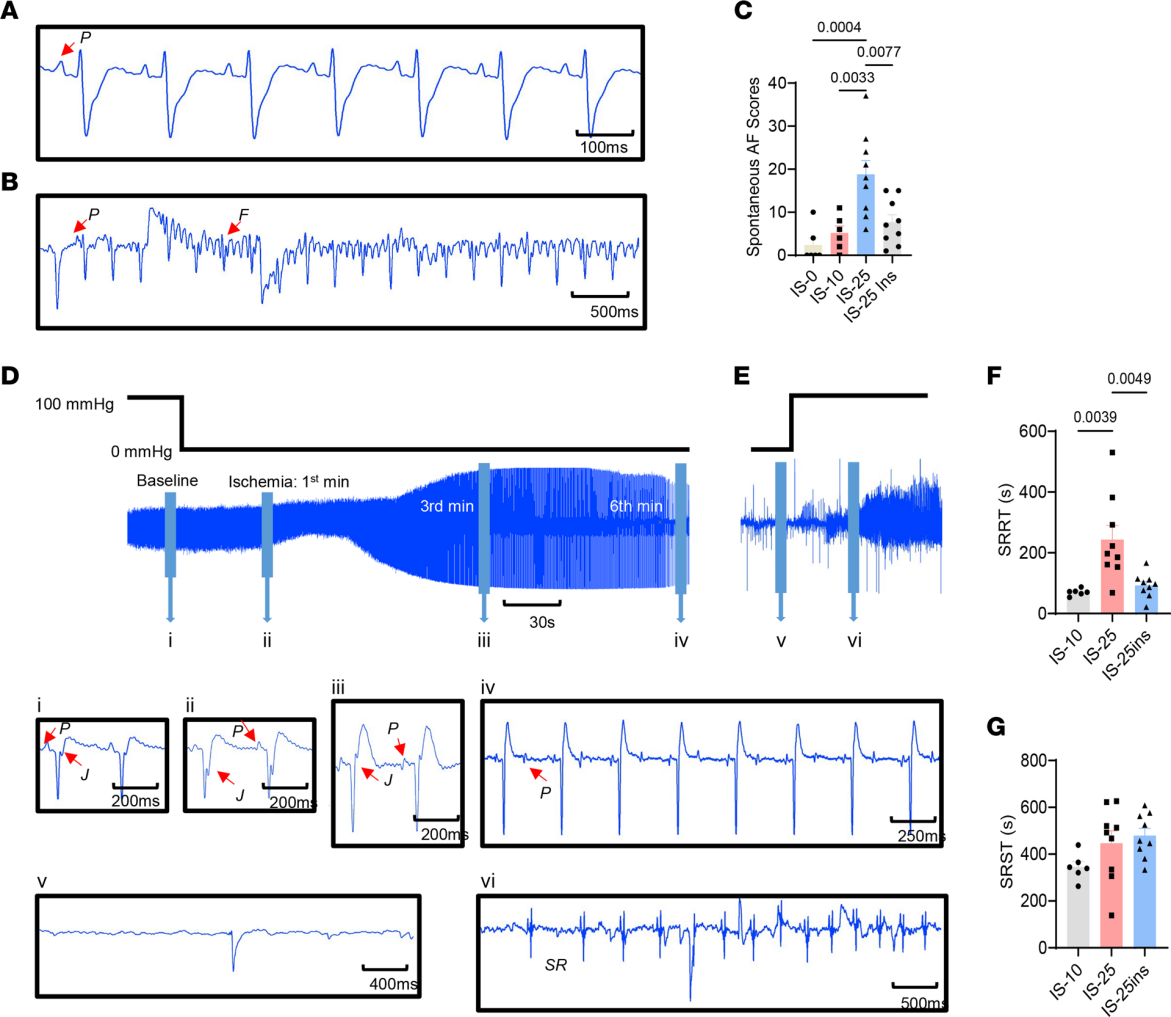
Insulin reduces the susceptibility to spontaneous AF and SRRT after reperfusion. (**A**) Sinus rhythm. P waves were clear and identifiable. (**B**) An episode of AF. P waves were replaced by f waves, which suggested an episode of AF. (**C**) Scores of the spontaneous AF during I/R. (**D** and **E**) ECG changes during perfusion. ECG at baseline (i), first minute of ischemia (ii), third minute of ischemia (iii), sixth minute of ischemia (iv), before reperfusion (v), and after reperfusion (vi). (**F**) SRST. (**G**) SRRT. Data were presented as mean ± SEM; *n* = 6, 6, 9, and 9 (biological repeats), respectively, in IS-0, IS-10, IS-25, and IS-25ins; statistical analysis was performed by 1-way ANOVA with Tukey’s multiple-comparison test.

Figure 6A shows a representative baseline electrocardiogram with clear and equal P waves, while Figure 6B shows a representative spontaneous AF where P waves disappeared and were replaced by f waves. A higher score was only found in the IS-25 group compared with IS-0, IS-10, and IS-25ins (*P* = 0.0004, 0.0033, and 0.0077, respectively, Figure 6C), suggesting that 25 minutes of ischemia increased susceptibility to spontaneous AF, but the use of insulin was protective. As shown in Figure 6, D and E, representative electrocardiograms display changes from baseline to cardiac arrest and recovery after ischemia, which include the (i) baseline, (ii) J point depression at the first minute of ischemia, (iii) J point and ST segment elevation at the third minute of ischemia, (iv) atrioventricular dissociation at the sixth minute of ischemia, (v) ventricular asystole before reperfusion, and (vi) sinus rhythm recovery after a certain period. There were no significant differences in SRST among ischemia conditions with or without insulin intervention. However, SRRT in 25 minutes of ischemia was significantly prolonged compared with 10 minutes of ischemia (*P* = 0.0039, Figure 6F), while with insulin, SRRT was shortened (*P* = 0.0049, Figure 6, F and G). These data support the conclusion that insulin provides protection against I/R-induced SAN dysfunction, aligning with the findings of SANRT after burst pacing.

### Insulin prevents I/R-induced change of transcriptome in the right atrium.

**Figure 7:**
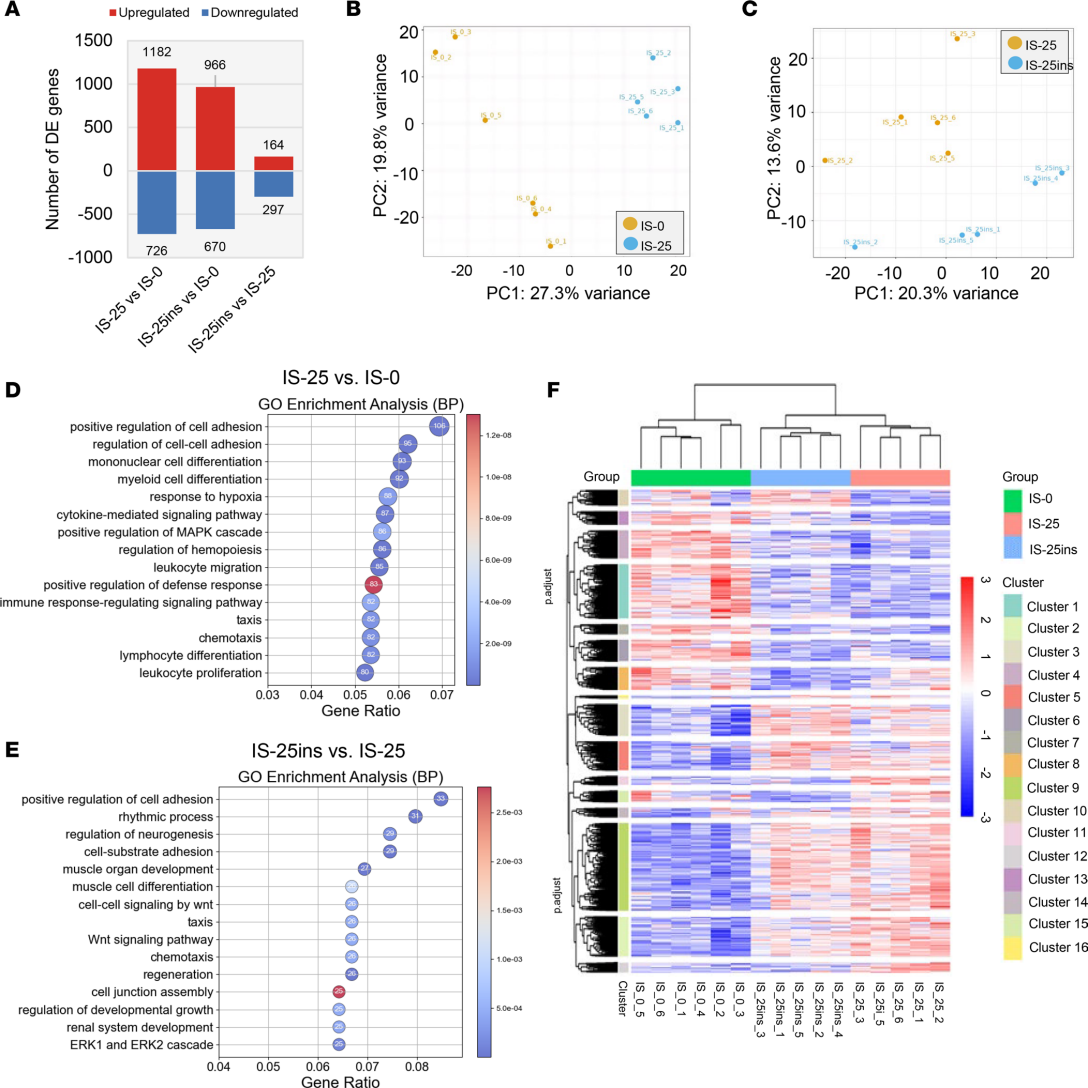
Gene expression and pathway analysis of right atrial myocardium. (**A**) Bar graph showing the number of DEGs between IS-0, IS-25, and IS-25ins groups. (**B** and **C**) Principal component analysis of gene expression across different groups (IS-0 vs. IS-25 in panel **B** and IS-25 vs. IS-25ins in panel **C**). (**D** and **E**) Dot plot of GO enrichment analysis across different groups (IS-0 vs. IS-25 in panel **D** and IS-25 vs. IS-25ins in panel **E**). (**F**) Cluster analysis of DEGs across the 3 groups. *n* = 6, 5, 5 (biological repeats) in IS-0, IS-25, and IS-25ins, respectively.

To investigate the mechanisms of POAF and the protective effects of insulin, SAN tissue from various groups was collected for bulk RNA sequencing. In the 25 minutes of ischemia group, 1,182 genes were upregulated and 726 genes downregulated (FDR < 0.05) compared with the nonischemia group. The IS-25ins group showed fewer differentially expressed genes (DEGs) than the IS-25 group, with 966 genes upregulated and 670 genes downregulated, respectively. Only 461 DEGs were identified when comparing IS-25ins with IS-25 (Figure 7A). Principal component analysis (PCA) demonstrated distinct clustering of the samples in the comparison between nonischemia and IS-25 groups, as well as between IS-25 and IS-25ins groups (Figure 7, B and C). The top 5 altered Gene Ontology (GO) pathways in the ischemia for 25 minutes versus nonischemia comparison included positive regulation of cell adhesion, mononuclear cell differentiation, myeloid cell differentiation, and response to hypoxia (Figure 7D). In the comparison between IS-25ins and IS-25, the predominant pathways were positive regulation of cell adhesion, rhythmic process, regulation of neurogenesis, cell-substrate adhesion, and muscle organ development (Figure 7E). I/R induced substantial gene expression changes, further exacerbated by insulin treatment in the positive regulation of cell adhesion GO term (Supplemental Figure 5). Kyoto Encyclopedia of Genes and Genomes (KEGG) analysis revealed alterations in the MAPK pathway (Supplemental Figure 6) and PI3K/AKT pathway (Supplemental Figure 7) in the IS-25 group compared with IS-0, with insulin partially reversing these changes, highlighting its role in modulating cell apoptosis and stress response. Additionally, cluster analysis categorized DEGs across the 3 comparisons into 16 distinct clusters (Figure 7F). Insulin treatment partially reversed gene expression changes in clusters 10–15 induced by I/R. In clusters 1–9 and 16, the gene expression patterns between the IS-25 and IS-25ins groups were similar (Supplemental Figure 8). Notably, clusters 11 and 12 were enriched in the circadian rhythm pathway, and cluster 15 was enriched in the ferroptosis pathway (Supplemental Data 1).

### Insulin does not substantially change the abundance of intermediates in the glucose metabolism pathway.

Tissues from right atrial myocardium of the IS-0, IS-25, and IS-25ins groups were collected for ion chromatography mass spectrometry (IC-MS), covering glycolysis, the TCA cycle, the pentose phosphate pathway, nucleotide metabolism, and other metabolic pathways. A total of 119 metabolites were detected and passed quality control. PCA revealed distinct clustering of the 3 groups (Supplemental Figure 9A). Forty-seven metabolites were significantly different between the IS-0 and IS-25 groups, with adjusted *P* values below 0.05; 16 were downregulated and 31 were upregulated in the IS-25 group (Supplemental Figure 9B). Insulin pre-perfusion in the IS-25ins group led to 19 downregulated and 11 upregulated metabolites compared with IS-25 (Supplemental Figure 9C). Pathway analysis showed that ischemia for 25 minutes followed by 30 minutes of reperfusion affected several pathways, including sucrose metabolism, fructose and mannose metabolism, alanine, aspartate and glutamate metabolism, the TCA cycle, glycolysis, pentose phosphate pathway, and others (Supplemental Figure 9D). Insulin pre-perfusion affected sucrose metabolism, taurine and hypotaurine metabolism, pentose phosphate pathway, fructose and mannose metabolism, alanine, aspartate and glutamate metabolism, amino sugar and nucleotide sugar metabolism, and glycolysis compared with IS-25 (Supplemental Figure 9E).

We analyzed the abundance of the metabolites in glucose metabolism to investigate if insulin affects glucose metabolism in the atrial myocardium. First, I/R in the IS-25 group resulted in decreased levels of glucose, glucose 6-phosphate, and fructose 6-phosphate and increased levels of glycerol 3-phosphate, 2-phosphoglycerate, and phosphoenolpyruvate compared with the IS-0 group. Insulin pretreatment in the IS-25ins group led to increased levels of glucose 1-phosphate, glucose 1,6-bisphosphate, glucose 6-phosphate, and fructose 6-phosphate compared with the IS-0 group, as well as increased levels of glycerol 3-phosphate compared with IS-25 (Supplemental Figure 10). These data implicate reduced glucose utilization in ischemic right atrial myocardium and increased glucose uptake induced by insulin. Second, neither I/R nor insulin pretreatment resulted in consistent changes of metabolites in TCA cycle and the levels of pyruvate and lactate (Supplemental Figure 10). Third, insulin treatment did not change ATP production in the ischemic atrial tissue (Supplemental Figure 11, A–C). Therefore, it is unlikely that altered glucose metabolism substantially contributed to insulin-mediated cardioprotection against ischemia-induced AF in the current study.

### Insulin reduced both cardiomyocyte and noncardiomyocyte apoptosis.

**Figure 8:**
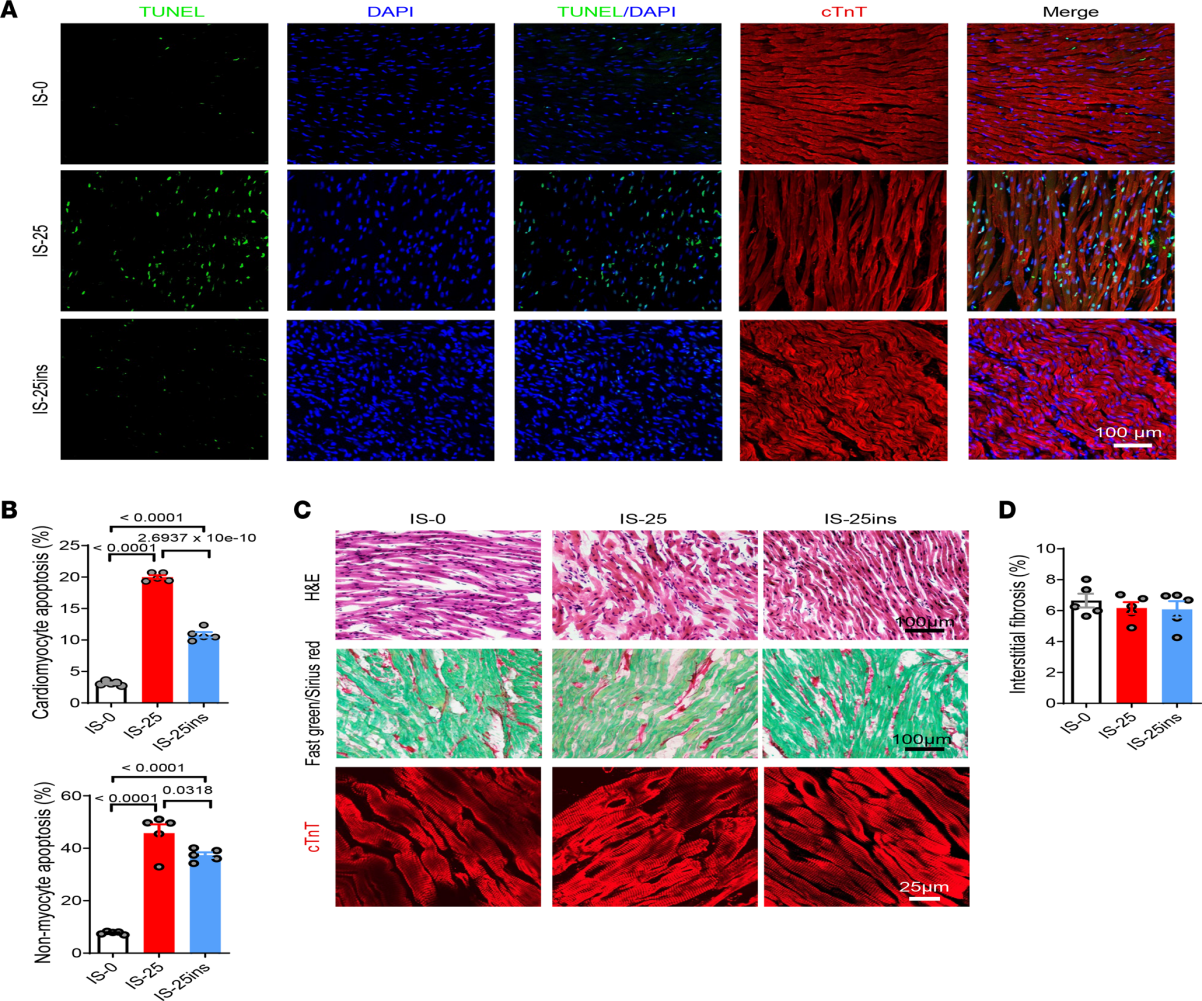
Insulin reduces cardiomyocyte apoptosis. (**A**) Cell apoptosis is measured by TUNEL staining. Cell nuclei were counterstained by DAPI. Bar = 100 μm. (**B**) Quantification of TUNEL positively staining nuclei of cardiomyocytes and nonmyocytes, respectively. The numbers of apoptotic cell nuclei were normalized to the total number of nuclei for cardiomyocytes and nonmyocytes, respectively. Data were represented as mean ± SEM. *n* = 5 (biological repeats) in each group. One-way ANOVA with Tukey’s multiple-comparison test. (**C**) H&E was performed to observe cardiac inflammation and gross morphology. Bar = 100 μm. Fast green/Sirius red staining was performed to determine the interstitial fibrosis. Bar = 100 μm. Immunostaining using antibodies against cardiac troponin T (cTnT) was performed to visualize cardiomyocyte morphology. Bar = 25 μm. (**D**) Quantification of interstitial fibrosis. Data were represented as mean ± SEM. *n* = 5 (biological repeats) in each group. One-way ANOVA with Tukey’s multiple-comparison test.

It was reported that cardiomyocyte apoptosis contributes to the pathogenesis of AF in patients and preclinical animal models (25, 26). Therefore, we measured cell apoptosis in right atrial heart tissue sections using terminal deoxynucleotidyl transferase dUTP nick end labeling (TUNEL) staining. Our data showed that ischemia for 25 minutes significantly increased apoptosis of both cardiomyocytes and noncardiomyocytes compared with nonischemic conditions, and treatment with insulin markedly reduced apoptosis of both cell types (Figure 8, A and B). There was no difference in the morphology of cardiomyocytes, sarcomere structure, and interstitial fibrosis between nonischemic and ischemic right atria regardless of insulin treatment, as depicted in Figure 8, C and D.

## Discussion

Although acute ischemia is thought to contribute to the development of POAF, the exact mechanisms remain unclear. Our study aimed to investigate the role of acute atrial I/R injury in the initiation and maintenance of AF and to understand the potential role of I/R in the pathogenesis of POAF as well as treatment options. Specifically, we observed that subjecting the entire atria to acute ischemia for 25 minutes followed by 30 minutes of reperfusion led to a substantial increase in RAF susceptibility (Figure 1), suggesting that the right atrium is more likely to develop AF than the left atrium under the same ischemic condition. Previous studies have emphasized the role of the left atrium in triggering AF. While the left atrium is the main source of blood clots and LAF is more common because of structural changes influenced by a higher prevalence of left ventricular diseases and hypertension, it is important not to overlook the significance of RAF. As the population ages and the rates of chronic lung diseases and right heart failure increase, the levels of remodeling and AF incidence in the right atrium are also on the rise (27). RAF itself can lead to LAF as there are no physical barriers between the 2 atria. In fact, some procedures targeting AF in the left atrium may be ineffective if the AF originates from the right atrium (28). Atrial infarction has variable incidence rates ranging from 0.7% to 42% in autopsy data, with the incidence of right atrial infarction being 5 times higher than that of the left atrium (29). Thus, our research highlights the importance of the right atrium in the development of POAF, as it is more prone to AF compared with the left atrium. It is important to note that in this study, we treated atrial flutter (AFL) the same as AF and scored them using the same standard. Distinguishing between triggered activities and AFL was difficult in our study because of the clear waveform and rhythm of atrial waves. However, patients with AFL may be at a higher risk of developing AF, as AFL can create conditions that promote AF through atrial electrical and structural remodeling (30–33).

The increased susceptibility may be attributed to the complex structure of the ischemic right atrium, which includes the dysfunction in the SAN, atrioventricular node, and autonomic nerves (34). Our optical mapping data showed that SAN plays a critical role in initiating activities in the macro-reentrant circuits (Figure 1 and Supplemental Figure 2), and dysfunction in the sinus node could increase susceptibility to AF (Figure 6). Furthermore, our data suggest that acute atrial ischemia–induced dysfunction in the SAN may be a focal origin of triggered activities that lead to AFL and AF in POAF (Figures 1 and 6). The 25-minute window of ischemia is relatively short, particularly considering that donor hearts undergo 4–6 hours of cold ischemia prior to transplantation (35). Prolonged warm ischemia time (WIT) has been associated with a poorer prognosis, with over 80 minutes of WIT linked to higher mortality rates (35, 36). Our experiment inducing whole-heart ischemia in a Langendorff model allowed us to study acute atrial ischemia and its effects on both atria, similar to WIT. The connection between AF onset and a 25-minute WIT window suggests that this period could be a critical risk factor for POAF, and reducing WIT may help prevent POAF. Our study revealed that acute atrial ischemia–induced RAF involves both reentry and triggered activities, with half of the triggered activities originating from and/or near the SAN (Figure 1). Therefore, addressing dysfunction in the SAN could prevent AF and POAF.

Currently, there is no efficient way to treat POAF. There has been debate over whether the use of GIK is effective in preventing POAF, but the current clinical data do not appear promising. A recent meta-analysis of 18 randomized controlled trials involving 2,000 patients showed that while GIK infusion during cardiac surgery reduced total mortality and the risk of postoperative myocardial infarction, it did not have a substantial impact on the prevalence of POAF (37). This was consistent with an earlier analysis that found GIK did not markedly reduce POAF in patients undergoing coronary artery bypass graft surgery (38). The difference between the expected outcomes and the actual results was attributed to factors such as patient selection, imperfect assessment of effects, inconsistent definitions of patient outcomes, and potential biases (39). More preclinical and clinical experiments are needed to further evaluate the impact of GIK on POAF following heart transplantation. Nevertheless, data from a meta-analysis have shown that perioperative use of insulin reduced the incidence of AF in patients undergoing cardiac surgery (23, 24). Furthermore, repeated insulin usage may help mitigate diabetes-related atrial fibrotic remodeling and the development of AF (40). These results emphasize the potential role of insulin as a preventive measure for POAF. Our data also demonstrated that insulin can prevent acute ischemia–induced AF. Administering insulin before ischemia prevented the occurrence of AF (Figure 2), suggesting that pretreatment with insulin before heart transplantation may help prevent the onset of POAF.

It remains unclear how acute activation of insulin signaling affects cardiac electrophysiology. Proper calcium handling contributes to stabilizing the cardiac APD and effective refractory period (ERP), which are essential for synchronized electrical activity throughout the heart, reducing the risk of triggered activity and reentrant circuits (41–43). Insulin has been shown to improve contractility in the hearts with I/R injuries by increasing AKT and sarcoplasmic reticulum calcium-ATPase, and PI3K/AKT signaling activated by the insulin receptor substrate further improves L-type calcium channel function by regulating channel translocation to plasma membrane (44–46). Conversely, the deletion of insulin receptors in cardiomyocytes, which leads to a lack of insulin signaling in the heart, can cause repolarization abnormalities. Additionally, basic studies on isolated sheep or canine hearts have shown that acute ischemia–induced AF is associated with altered APD because of impaired function of ATP-sensitive potassium channel and the reentrant activities around the infarct border zone (6, 47). Impaired insulin signaling has been linked to decreased expression of the fast component of the transient outward K^+^ current (Ito/fast), which results in a reduction of Ito/fast and ultimately leads to prolongation of APD (48). Insulin treatment increases potassium uptake, aiding in restoring sinus rhythm and stabilizing the ischemic myocardium (23). Furthermore, a recent study demonstrated that insulin/insulin-like growth factor 1 signaling plays a crucial role in maintaining SAN function. Loss of insulin receptors can cause a slow heart rate and downregulate the expression of hyperpolarization activated cyclic nucleotide gated potassium channel 1 and 4 (49). In the current study, insulin treatment time is rather short (i.e., up to 85 minutes; Figure 1). It is unlikely the cardioprotection of insulin is through the changed expression of ion channels. However, our data suggest that prolonged ischemia reduces APD, ERP, and conduction velocity, while prolonging calcium transient in the right atrium. Insulin pretreatment before ischemia prevented ischemia-induced electrophysiological disorders (Figures 3 and 4). Therefore, we speculate that the observed insulin-mediated cardioprotection may occur by regulating calcium transient, by reducing reentry, and by triggered activities in the ischemic SAN.

The molecular mechanisms that underlie insulin-mediated anti-AF effects remain unclear. In this study, the protective effect of insulin was also observed when glucose was replaced by pyruvate along with insulin (Figure 2) or by 2DG (an inhibitor of glucose metabolism). Metabolomic analyses indicated that insulin did not substantially change pyruvate and lactate levels in right atrial myocardium (Supplemental Figures 9 and 10). Under normal circumstances, the heart primarily uses fatty acids as fuel, producing 60%–90% of ATP, with 10%–40% coming from glucose (50). Our study was conducted in a Langendorff perfusion system with only glucose or pyruvate as an extracellular energy source, while β-oxidation of fatty acids was completely inhibited. Based on our data, it is unlikely that the mechanism underlying insulin-mediated cardioprotection involved changes in glucose or fatty acid metabolism. RNA-sequencing data indicated that both I/R and insulin pretreatment resulted in changes in gene expression in the pathways of cell apoptosis and adhesion (Figure 7). KEGG analysis revealed that insulin prevented I/R-mediated changes in the expression of genes in the MAPK and PI3K/AKT pathways (Supplemental Figures 6 and 7), which are crucial for regulating cell apoptosis and stress response (51). TUNEL staining data showed that insulin prevents I/R-induced apoptosis of both cardiomyocytes and nonmyocytes in the right atrial myocardium, without substantial altering of sarcomere structure or extent of interstitial fibrosis (Figure 8). Future studies are needed to comprehend the precise molecular mechanism for insulin-mediated regulation of cardiomyocyte electrophysiology.

Our study has several limitations. First, the long-term effects of insulin on the delayed onset of AF remain unclear. Second, in vivo studies were not included in this research. In vivo investigations of insulin’s effects on regional atrial ischemia are necessary to confirm the findings in a more clinically relevant context, such as chronic ischemia–related AF caused by microcirculation dysfunction. Third, further research is needed to determine if an insulin-enriched cardioplegia preservation solution could prevent the development of POAF after heart transplantation in larger animals or humans. Addressing these issues in future studies will offer more comprehensive insights and enhance the clinical applicability of the findings. Last, the insulin dose (5.0 mU/L) selected for this study was based on the study by Jonassen et al. (52). Additional studies are required to optimize the insulin dosage.

In conclusion, our study highlights the role of acute atrial ischemia as a preconditioning factor in the development of POAF, partly due to sinus node dysfunction. This discovery suggests that sinus node dysfunction may contribute to AF, offering a potential explanation for its onset. The protective effect of insulin against AF is only effective when administered before atrial ischemia. Additionally, the potential benefits of insulin in preventing POAF, such as using insulin-supplemented cardioplegia or GIK during heart transplantation, require further validation. Cardiac surgery or coronary interventional procedures are commonly performed in clinics to treat patients with congenital or acquired heart diseases. However, many cardiac procedures are associated with transient myocardial ischemia (6–8). Around 20%–30% of POAF episodes occur in patients during or shortly after cardiac surgery or coronary procedures (1, 3–5). Currently, there is no effective therapy to treat POAF. This study found that insulin has cardioprotective effects to prevent I/R-induced AF by stabilizing electrophysiology and reducing apoptosis. These findings could have important clinical applications by introducing potential approaches to improve heart preservation before transplantation or in nontransplant cardiac surgery. Additionally, locally applying insulin to the atria may offer protection during cardiac catheterization.

## Methods

### Sex as a biological variable.

Our study examined male and female animals, and similar findings are reported for both sexes.

### Experimental animals.

Both male and female Sprague-Dawley rats (8~12 weeks, 250–300 g) were purchased from Charles River Laboratories. Animals were anesthetized by ketamine and xylazine (100 mg and 10 mg/kg, respectively), heparinized (200 IU), and euthanized by heart removal. Each heart was immediately stored in cold cardioplegia (contains NaCl 110 mM, CaCl_2_•2H_2_O 1.2 mM, KCl 16 mM, MgCl_2_ 16 mM, NaHCO_3_ 10 mM) and cannulated for Langendorff perfusion with modified Tyrode’s solution, which contains NaCl 130 mM, KCl 4 mM, CaCl_2_•2H_2_O 1.8 mM, MgCl_2_ 1 mM, NaH_2_PO_4_ 1.2 mM, NaHCO_3_ 20 mM, and glucose 5.6 mM.

### Study design.

The group setup is depicted in Figure 1A and Figure 2A. Ischemia-induced myocardial injury can occur as early as 10 minutes (53) and is irreversible if the ischemia lasts longer than 25 minutes (54). Therefore, the impact of ischemia for 10 or 25 minutes on atrial electrophysiology was tested in this study. First, ischemia was induced for 0, 10, or 25 minutes (referred to as IS-0, IS-10, and IS-25, respectively) to compare the effects of different durations of ischemia on AF. Second, the effectiveness of insulin was examined by adding insulin (5 mU/mL) to Tyrode’s solution with regular glucose at 5.6 mM (referred to as IS-25ins). Third, to determine if the protective effect depended on glucose, a double dose of glucose (11.2 mM, without insulin, referred to as 11.2GLU), replacement of glucose with 5.6 mM pyruvate (PRV, or with insulin, referred to as PRV-insulin), or 2DG with insulin was used. It is important to note that these different energy substrates were perfused throughout the entire procedure, except during the ischemia phase. However, the therapeutic effects may have continued because the substrates were not washed out. Fourth, insulin was administered for only 30 minutes during reperfusion (referred to as Insulin^-reperfusion^) to determine if insulin still provided protection after ischemia. A total of 78 rats were used in this study, and a group size of at least 6 was determined based on previous publications with similar research design (52, 55, 56).

### Ex vivo heart Langendorff perfusion and open atrial endocardial optical mapping.

This procedure was adopted to induce conditional ischemia, conduct drug intervention, and perform optical mapping as shown in Supplemental Figure 1 following instructions previously described (56, 57). Perfusion pressure was maintained at 80–120 mmHg while temperature remained at 37°C and pH at 7.35 ± 0.05. ECG was continuously recorded by a bio-amplifier (ADInstruments) at a lead II direction. Blebbistatin (15 μmol/L; BML-EI315-0025, Enzo Life Sciences, Inc.) was supplemented 10 minutes after heart stabilizing with Tyrode’s solution and present throughout the entire procedure to reduce the influence of motion artifact on imaging. Zero-flow ischemia was induced by shutting off the perfusion while temperature remained at 37°C followed by 30 minutes of reperfusion. Then, RH237 (1 μmol/L in 1 mL Tyrode’s solution; S1109, Thermo Fisher Scientific) was injected into the perfusion pipeline for 10 minutes to allow for optical mapping of action potential. Both ventricles were removed. Atria were dissected and anchored to form a superficial perfusion with a pair of pacing leads at the edge of the right or left atrium to allow programmed electrical stimulation (Labchart, ADInstruments).

The SAN region is located near superior vena cavae and the coronary sinus, as confirmed via optical voltage mapping using the site of the initial depolarization. S1S1 pacing at different PCLs (150, 130, 120, 110, 100, 90, 80, and 70 ms) and ERP (with extra stimuli S1-S4) and AF vulnerability (by 50 Hz burst pacing) were tested. SANRT was measured after a burst pacing based on optical mapping of action potential of SAN as shown in Figure 1, H–J. The imaging settings of the optical mapping can be seen in Supplemental Figure 1B. We equipped a green light source (530 nm, LEX3G, SciMedia Ltd.), excitation filter (520 nm long pass, Edmund Optics Inc.), dichroic mirror (edge at 560 nm), emission filter (580 nm or 600 nm long pass), and 1.0× objective lens and 85 mm camera lens, which forms a field of view at 16.5 × 16.5 mm, and a high-resolution optical mapping system that enables imaging 256 × 256 pixel signals (MiCAM03, SciMedia Ltd.). Data acquisition and analysis used the BV Workbench v.4 (SciMedia Ltd.). In addition, calcium transient signal was imaged using Rhod-2 AM (R1244, Thermo Fisher Scientific) as previously described (56). Typically, 20 μL RH237 stock solution (2.5 mg/mL) or Rhod-2 AM stock solution (1 mg/mL, plus 20 μL pluronic F-127, P3000MP, Invitrogen, Thermo Fisher Scientific) was diluted in 1 mL Tyrode’s solution and pulse injected to perfusion tubing around 10 minutes.

Data acquisition and analyses were performed with BV Workbench. Background noise was filtered using built-in software, including the applying of the mean/medium filters with frequency cutoff at 40 Hz. APD50, APD90, and CaTD80 were analyzed at different PCLs. The pixel size was 0.064 mm. A filter size of 25 × 25 was used to generate data on action potential, conduction velocity, or calcium transient. The SD value of conduction velocity was used to express the conduction heterogeneity. PCL at 130 ms was relevant to a physiological heart rate of rats, and conduction velocity at this PCL was quantified for conduction heterogeneity.

To perform leading region analysis in optical mapping, background noise was filtered, and the leading region in each cardiac cycle was identified with the application of leading region analysis in the software. Poincaré plots were used to visualize the variability and dispersion of these leading regions over time. The standard deviations (SD1 and SD2) were computed, and the SD_mean_ was calculated to quantify the overall dispersion of the leading regions. The Poincaré plot is an invaluable tool for analyzing time series data, particularly for assessing heart rate variability. Therefore, it was used for visualizing the dispersion of the leading regions. By plotting consecutive data points against each other, this method visualized the relationship between successive intervals, revealing patterns in short-term (SD1) and long-term (SD2) variability.

### Spontaneous AF scoring.

Spontaneous AF and flutter during ischemia and reperfusion were measured using an arrhythmia scoring system. The scoring system was as follows: 0 for no occurrence, 1 for duration less than 2 seconds, 2 for duration of 2 to less than 15 seconds, 3 for duration of 15 to less than 30 seconds, 4 for duration of 30 to less than 60 seconds, 5 for duration of 60 to less than 120 seconds, and 6 for duration of 120 seconds or longer. Additionally, the SRST after ischemia and the SRRT after reperfusion were recorded to evaluate the function of the SAN.

### Histology.

Atrial tissue was embedded in OCT compound and sectioned at 10 μm of thickness. A series of heart sections were used for H&E and fast green/Sirius red staining, and fibrosis was quantified as previously reported (58, 59). A commercial TUNEL kit (Thermo Fisher Scientific, C10617) was used to evaluate cell apoptosis rate in the right atrial myocardium. The fragmented cellular DNA indicating cell apoptosis was stained and visualized as GFP signal. Cardiomyocytes were costained by using a troponin T antibody (1:100, Abcam, ab45932) and labeled with Alexa Fluor 647 (1:250, Invitrogen, Thermo Fisher Scientific, A-31573). DAPI was used to visualize the cell nuclei. Images were taken with an Olympus IX83 microscope system or ZEISS confocal imaging system (LSM 800) and analyzed with Fiji ImageJ. The apoptosis levels of cardiomyocytes and noncardiomyocytes were analyzed.

### RNA sequencing.

RNA was isolated from ex vivo rat cardiac tissues and sent to Novogene for sequencing. Quality control and library preparation of RNA were conducted according to Novogene’s standard pipeline. Paired-end raw reads were sequenced on an Illumina platform. Raw RNA-sequencing reads were processed through Novogene’s bioinformatics analysis pipeline. Briefly, fastp (60) was used to remove adapter sequences and assess read quality. The splice-aware program Hisat2 (61) v2.0.5 was used to align reads to the *Rattus norvegicus* genome version 6.0 with Ensembl v102 annotation. Novel transcripts were predicted using StringTie (62) v1.3.3b. FeatureCounts (63) v1.5.0-p3 was used to obtain raw and normalized (fragments per kilobase of transcript per million mapped reads, FPKM) read counts. Pairwise differential expression analyses were conducted using raw counts with edgeR (64) v4.0.16. Genes were considered differentially expressed with FDR < 0.05. Ensembl IDs of DEGs were compared with Venn diagrams using Venny 2.1.0. GO and KEGG enrichment analyses were performed using clusterProfiler (65), and results were visualized using the R package ggplot2 v3.5.1. Pathways were considered significant at FDR < 0.05. Heatmaps of DEGs within each GO or KEGG term were generated using the R package pheatmap v1.0.12. Analysis of canonical pathways was conducted with the Ingenuity Pathway Analysis (IPA) software (Ingenuity Systems, QIAGEN). To compare IPA-derived pathway *z* scores — representing activation or repression — across different gene sets (i.e., pairwise differential expression analyses or subsets of genes from the Venn diagram), IPA Comparison Analysis and the R package pheatmap were used. To visualize global trends in gene expression and identify groups of genes with similar expression patterns, hierarchical clustering analysis was performed using DEGs across all comparisons. The hclust package in R was used to generate dendrograms of genes based on *z* score of log2(FPKM+1) using the complete linkage method. Genes were separated into 16 clusters to best identify groups of genes with similar expression pattern in nonischemic and insulin-treated ischemic tissues but with opposite expression pattern in ischemic tissues.

### IC-MS.

The IC-MS was performed at the MD Anderson Cancer Center Metabolomics Facility. Metabolites were extracted using ice-cold 0.1% ammonium hydroxide in 80:20 (v/v) methanol/water. After centrifugation at 17,000*g* for 5 minutes at 4°C, the supernatants were transferred to clean tubes and evaporated to dryness under nitrogen. The dried extracts were reconstituted in deionized water, and 5 μL of each sample was injected for IC-MS analysis. For IC, the mobile phase A (weak) was water, and mobile phase B (strong) was water containing 100 mM KOH. The system used was a Thermo Fisher Scientific Dionex ICS-5000+ with a Thermo Fisher Scientific IonPac AS11 column (4 μm particle size, 250 × 2 mm) maintained at 30°C, while the autosampler was chilled to 4°C. A gradient from 1 mM to 100 mM KOH was applied at a flow rate of 350 μL/min over a 60-minute total run time. Methanol was introduced using an external pump to enhance desolvation and sensitivity.

### Statistics.

Data were presented as mean ± SEM. For data compliance with the normality and homogeneity of variances, 1-way or 2-way ANOVA with Tukey’s multiple comparisons was used to compare difference among groups. Data for the AF duration (Figure 2C) were not compliant with the normality and homogeneity of variances and were analyzed with Kruskal-Wallis test followed by Dunn’s multiple comparisons test. A 2-tailed Student’s *t* test was used to compare difference between 2 groups in Figure 4. *P* < 0.05 suggested statistical difference. Graphs were generated with GraphPad Prism v10. For the IC-MS analysis (Supplemental Figure 10), 1-way ANOVA with Tukey’s multiple comparison was performed on all sample pairs. Metabolites with *P* < 0.05 were considered significant and identified as differential metabolites. Pathway analysis was performed using MetaboAnalystR 4.0 (66).

### Study approval.

All animal protocols were approved by the Institutional Animal Care and Use Committee (IACUC) of Mayo Clinic. All animal surgical procedures and euthanasia were performed based on approved IACUC protocol and in accordance with the National Institutes of Health (NIH) *Guide for the Care and Use of Laboratory Animals* (National Academies Press, 2011).

### Data availability.

All data associated with this study are present in the paper or the supplemental information, and raw data are included in the Supporting Data Values file. Reagents and materials associated with this study are available from the corresponding author. Python was used to calculate the SD1 and SD2 values in this study. The code can be retrieved from GitHub (https://github.com/LukeHQ-byte/Poincare-plot-SD1-SD2; commit ID dfc64ae). The RNA-sequencing data have been deposited in the NIH National Center for Biotechnology Information Gene Expression Omnibus database (accession number: GSE279356), and the metabolomics data have been deposited in NIH National Metabolomics Data Repository (study ID: ST003519).

## Author contributions

HQ initiated the project, conducted experiments, acquired and analyzed data, and wrote the manuscript. FL conducted experiments, acquired and analyzed data, and wrote the manuscript. HP and APG analyzed data. MR designed research studies and wrote the manuscript. WZ designed research studies, wrote the manuscript, and handled finance.

## Supplementary Material

Supplemental data

Supplemental data set 1

Supporting data values

## Version 1 11/14/2024

In-Press Preview

## Version 2 01/09/2025

Electronic publication
