# Supplementary material for: Insulin mitigates acute ischemia–induced atrial fibrillation and sinoatrial node dysfunction ex vivo
Source: JCI Insight. 2024 Nov 14;10(1):e185961. doi: 10.1172/jci.insight.185961 (PMC11721304; doi:10.1172/jci.insight.185961)
Supplement: Supplemental data [file jciinsight-10-185961-s260.pdf]

SUPPLEMENTAL MATERIALS

Insulin mitigates acute ischemia induced atrial fibrillation and sinoatrial node dysfunction

Huiliang Qiu,[1] Fan Li,[1] Hannah Prachyl,[1] Alejandra Patino-Guerrero,[1] Michael Rubart,[2], Wuqiang Zhu,[1]

[1]Department of Cardiovascular Medicine, Physiology and Biomedical Engineering, and Center for Regenerative Biotherapeutics, Mayo Clinic Arizona, Scottsdale, AZ 85259

[2]Department of Pediatrics, Wells Center for Pediatric Research, Indiana University School of Medicine, Indianapolis, IN 46202

Supplemental Materials include:

Supplemental figures 1-11 and figure legends

Supplemental tables 1-2

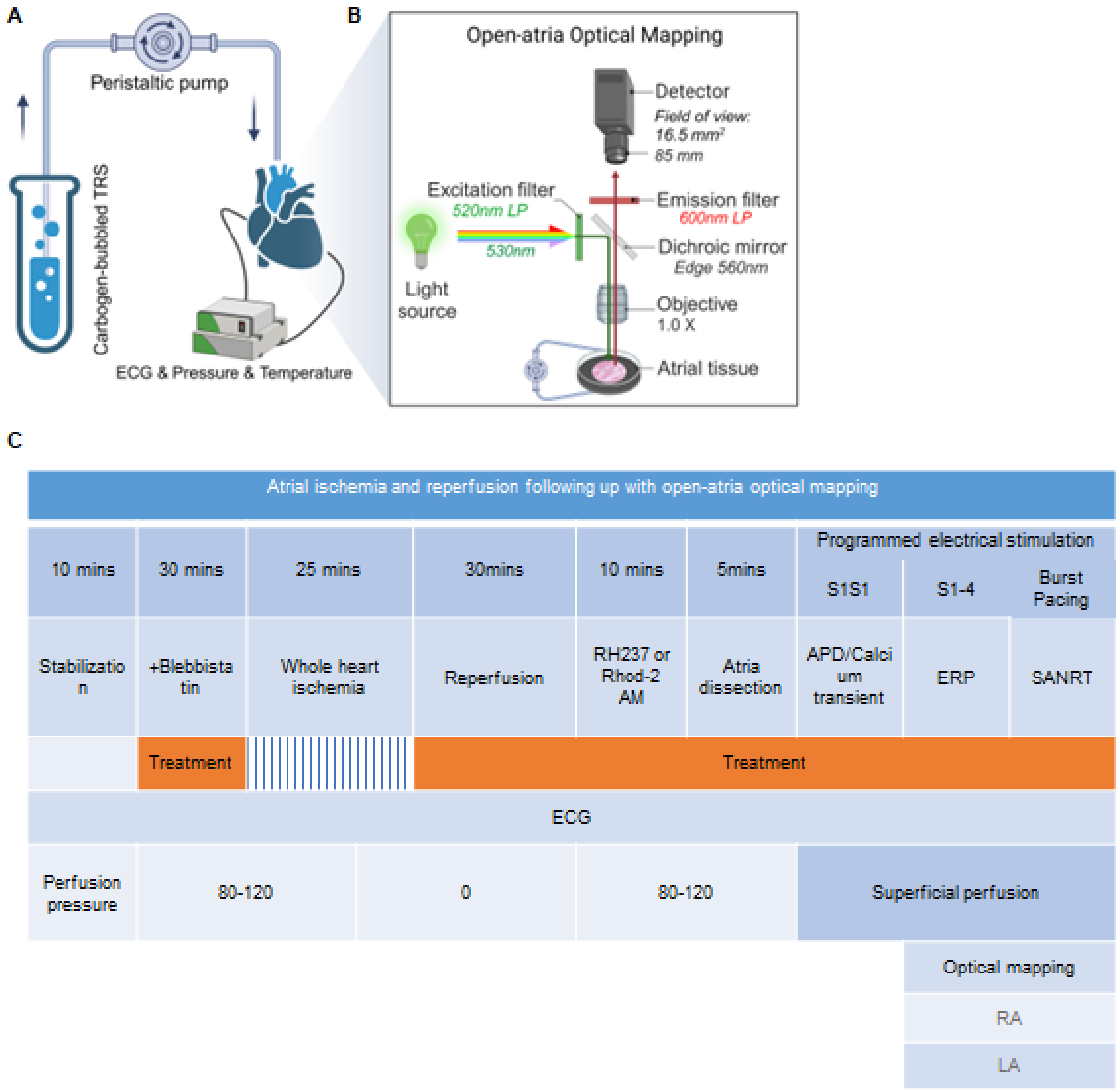

Supplementary Figure 1. Depiction of ex vivo Langendorff heart perfusion and open-atria optical mapping. (A) Isolated heart was perfused with modified Tyrode's solution while mean perfusion pressure was maintained at around 100 mmHg and temperature at 37 ± 0.5 ºC. **(B)** The optical mapping setting for assessing action potential with voltage sensitive dye, RH237, or calcium transient, with the Rhod-2 AM. A green light source (530 nm, LEX3G) was adopted. The excitation filter allows LED light longer than 520 nanometers (nm) to pass; LP indicates long-pass. Dichroic mirror with cutting edge at 560 nm reflects laser shorter than 560 nm and passes laser longer than

560 nm. Emission light will be further filtered with a 600 nm LP filter. The combination of objective and camera lens forms a field of view at 16.5 mm × 16.5 mm. **(C)** After heart stabilizing, blebbistatin was added into and maintained in Tyrode's solution the whole procedure. Perfusion closure was made to stop the perfusion so as to mimic conditions of ischemia for 0 minute, 10, or 25 minutes. Then reperfusion was allowed for 30 minutes before RH237 or Rhod-2 injecting. Open-atria dissection was operated to allow optical mapping of the endocardium or SAN. Programmed electrical stimulation including S1S1, extra stimuli, and 2-second 50 Hz bursting pacing was delivered.

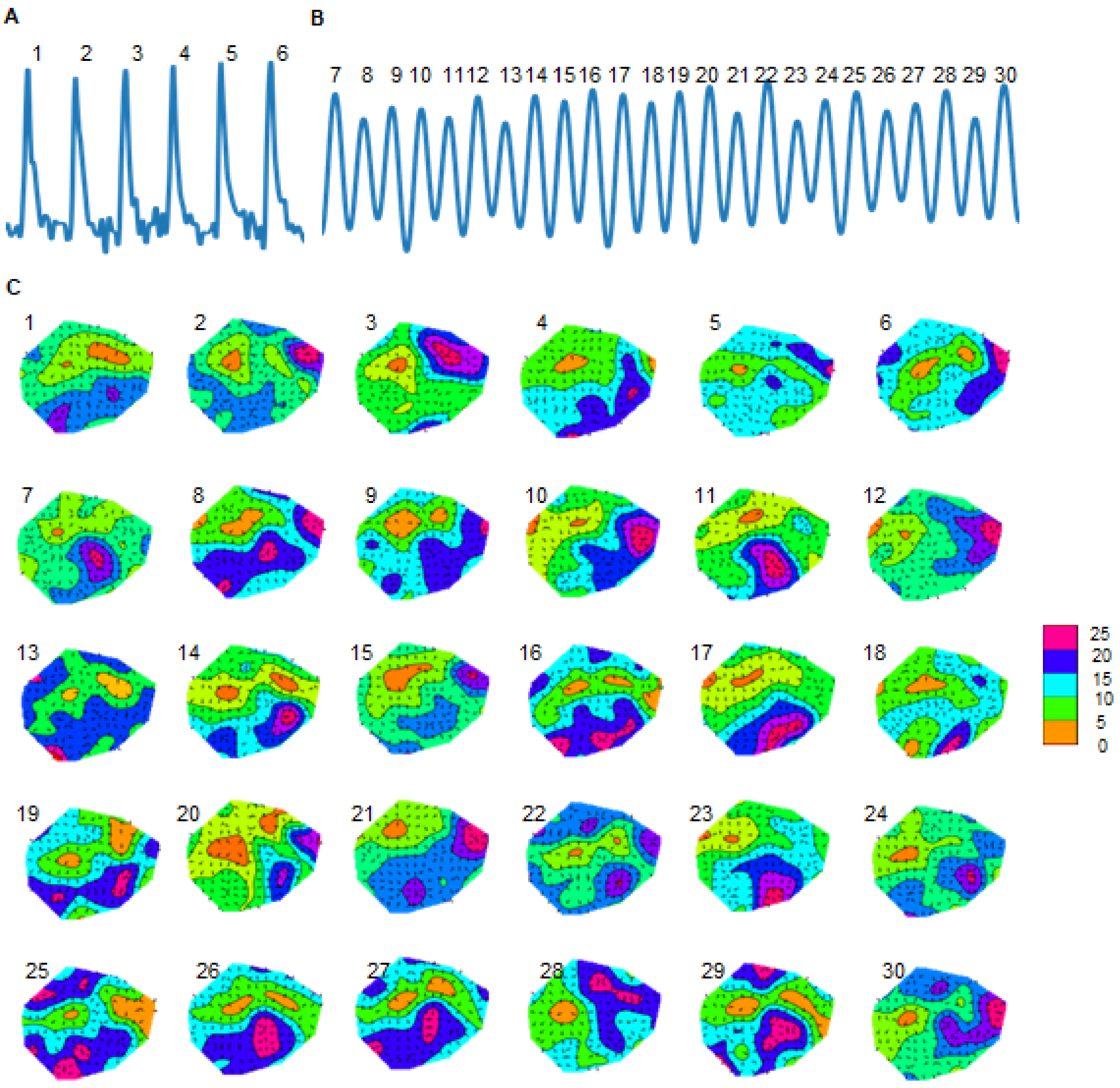

Supplementary Figure 2. Triggered activities originated from sinoatrial node (SAN) area. (A, B) Baseline action potential **(A)** and an AF episode **(B)** in ECG. **(C)** Representative activation maps. C1-6 represent the corresponding action potential at baseline in Panel A #1-6, showing the location of SAN. C7-30 represent the corresponding action potential in Panel B #7-30, showing the originated regions which are the same as SAN area. This data indicates the AF episode is originated from SAN.

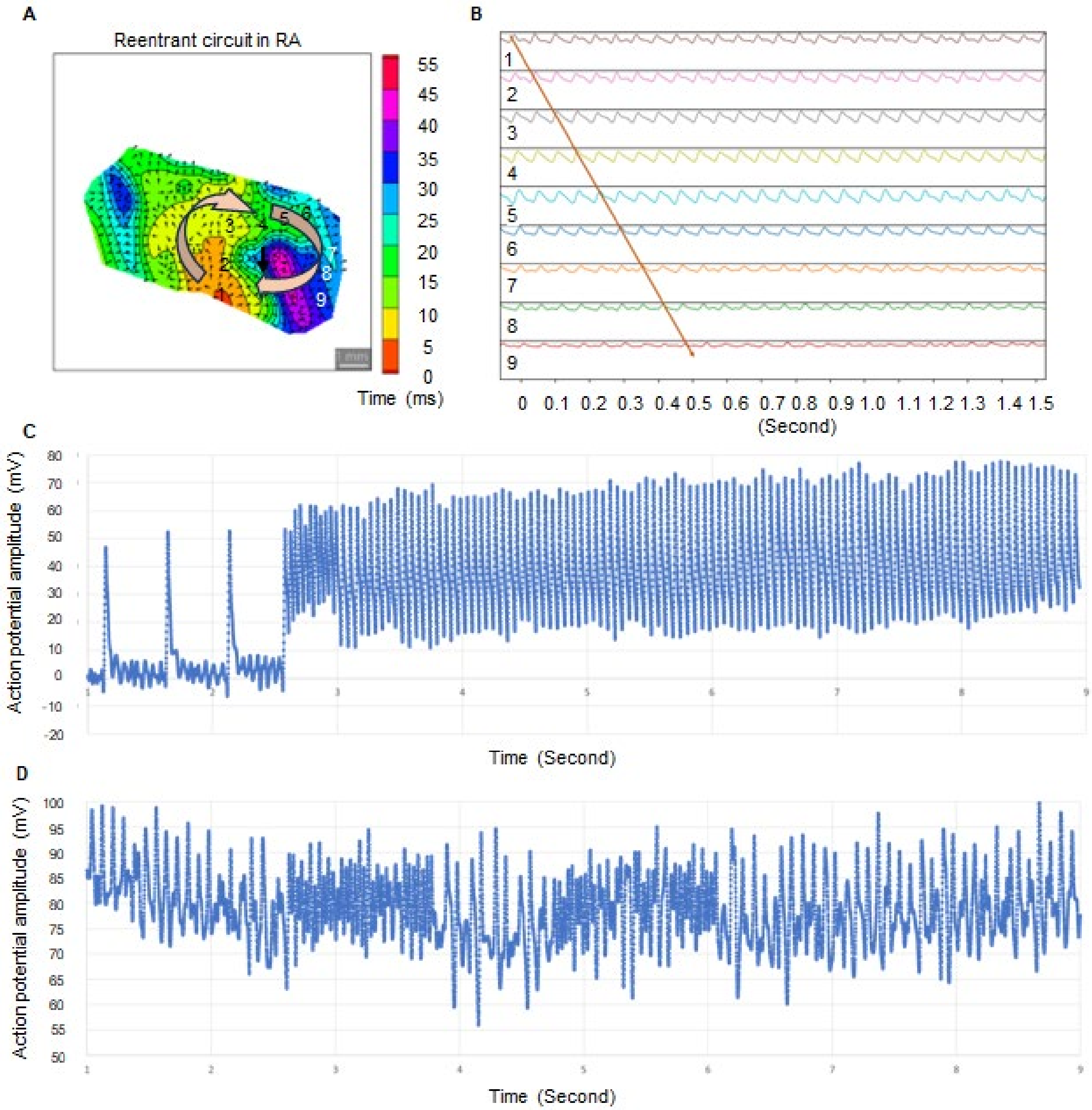

Supplementary Figure 3. Reentry mechanism of RAF. (A) A typical macro reentrant circuit. **(B)** The sequency of electrical conduction corresponding to the locations in panel A. The line represents the sequence of activation. Each segment in x-axis represent 0.1 second. **(C-D)** The induced arrhythmia shows relatively regular rhythm at early stage **(C)** but turns into chaotic rhythm at later stage **(D)**.

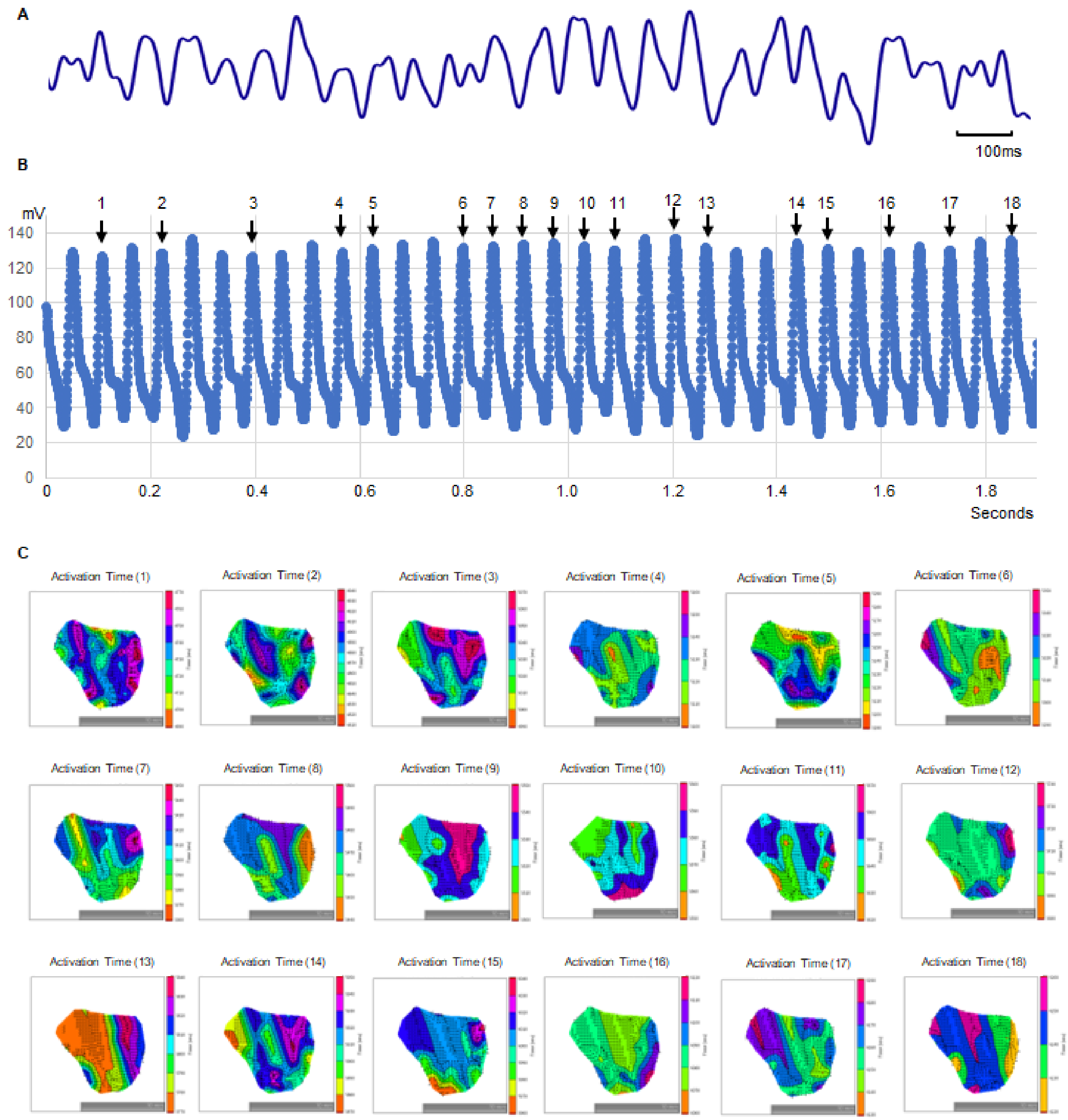

Supplementary Figure 4. (A) An AFL-like episode in ECG. **(B)** The activation maps in optical mapping.

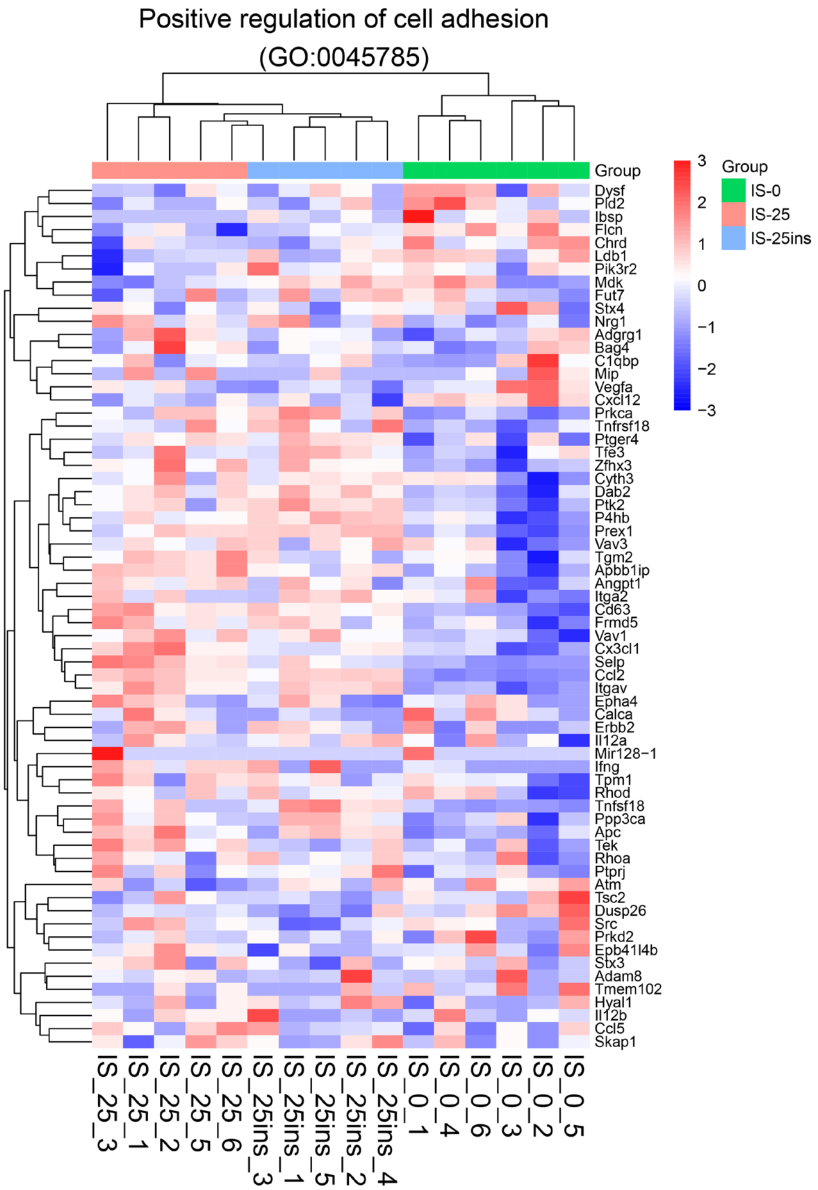

Supplemental Figure 5. Heat map of genes in the positive regulation of cell adhesion GO term. n=6 in IS-0 group, n=5 in IS-25 and IS-25ins groups, respectively.

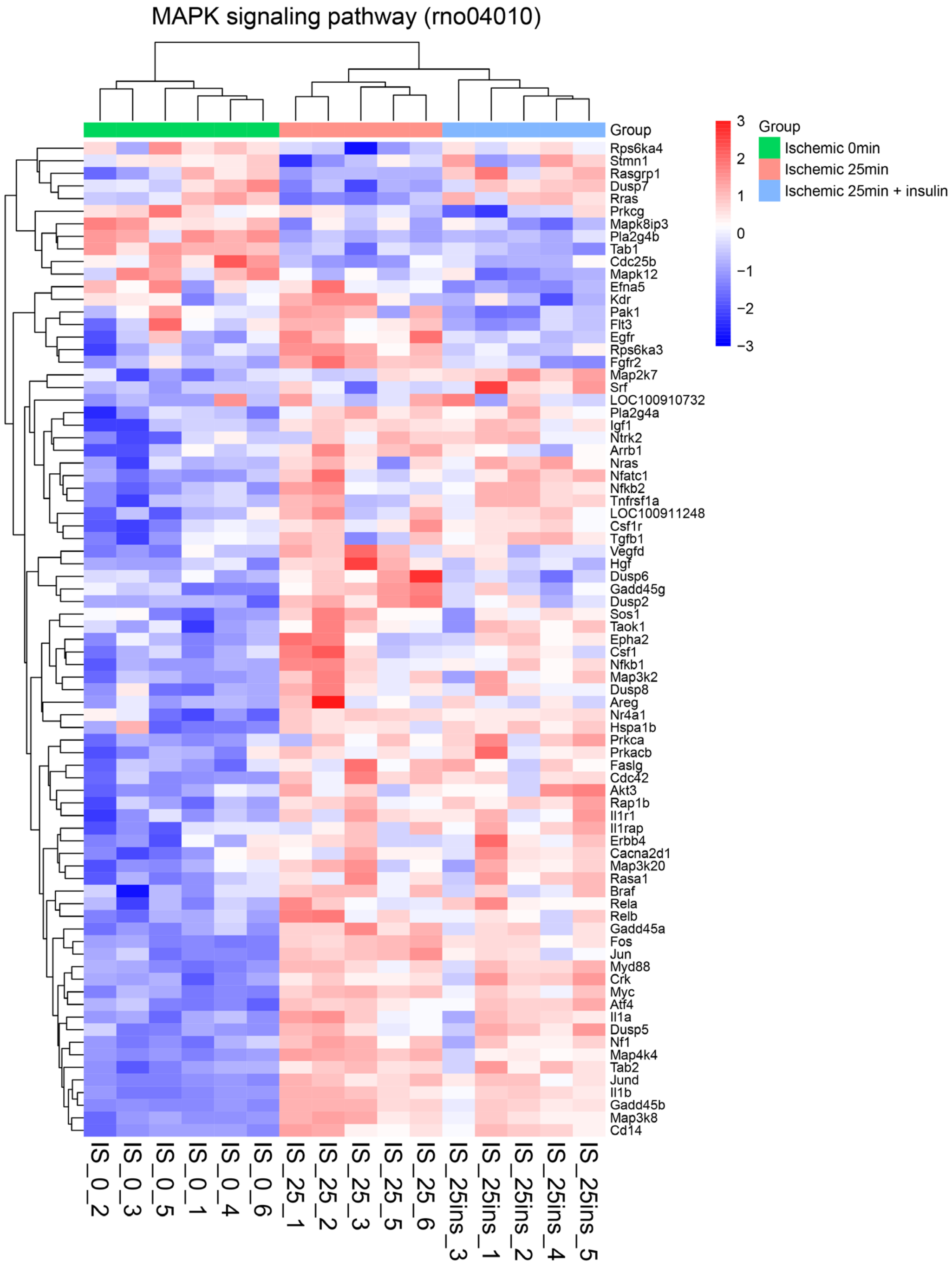

Supplemental Figure 6. Heat map of differentially expressed genes in the MAPK pathways. n=6 in IS-0 group, n=5 in IS-25 and IS-25ins groups, respectively.

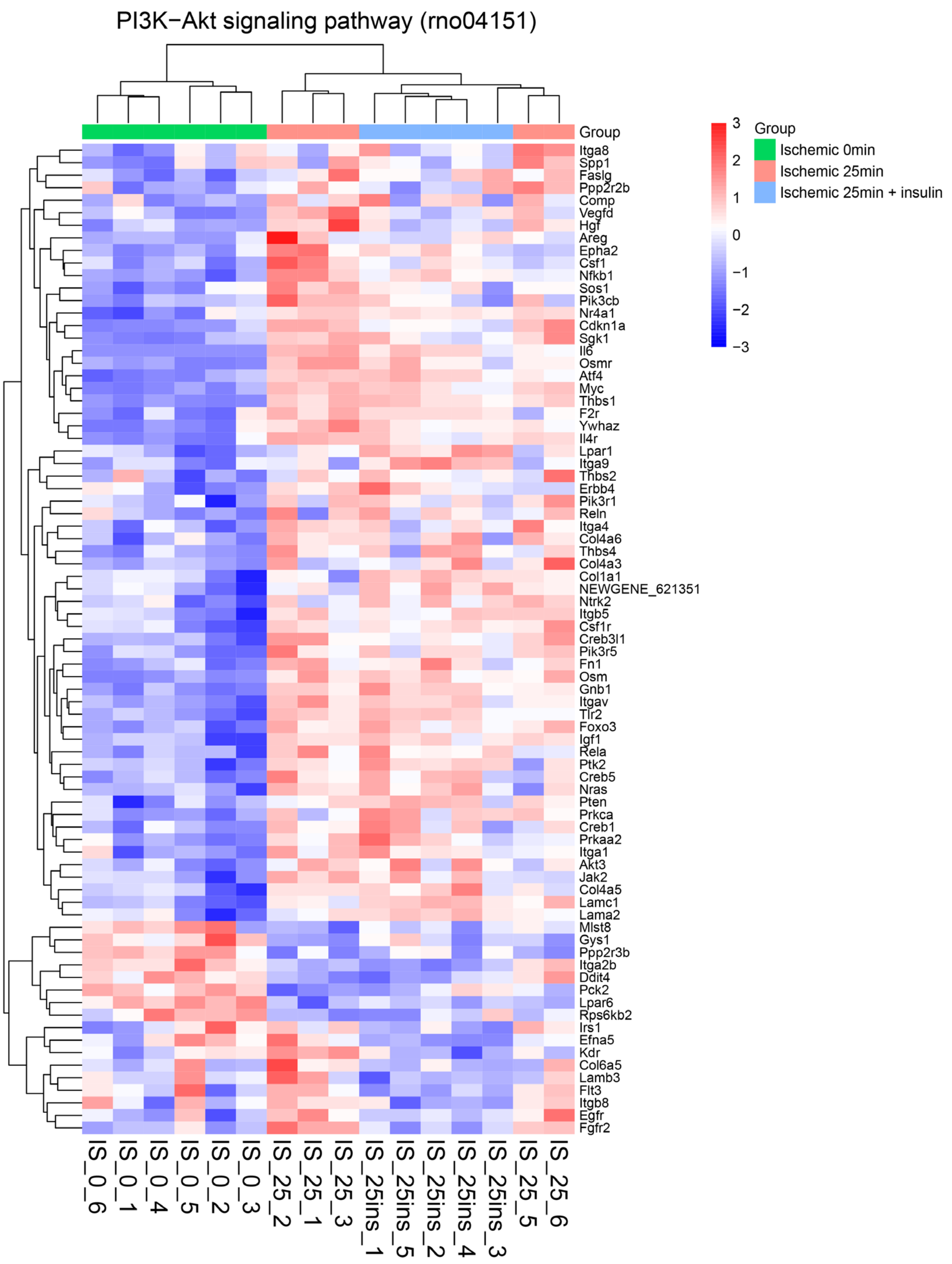

Supplemental Figure 7. Heat map of differentially expressed genes in the MAPK pathways. n=6 in IS-0 group, n=5 in IS-25 and IS-25ins groups, respectively.

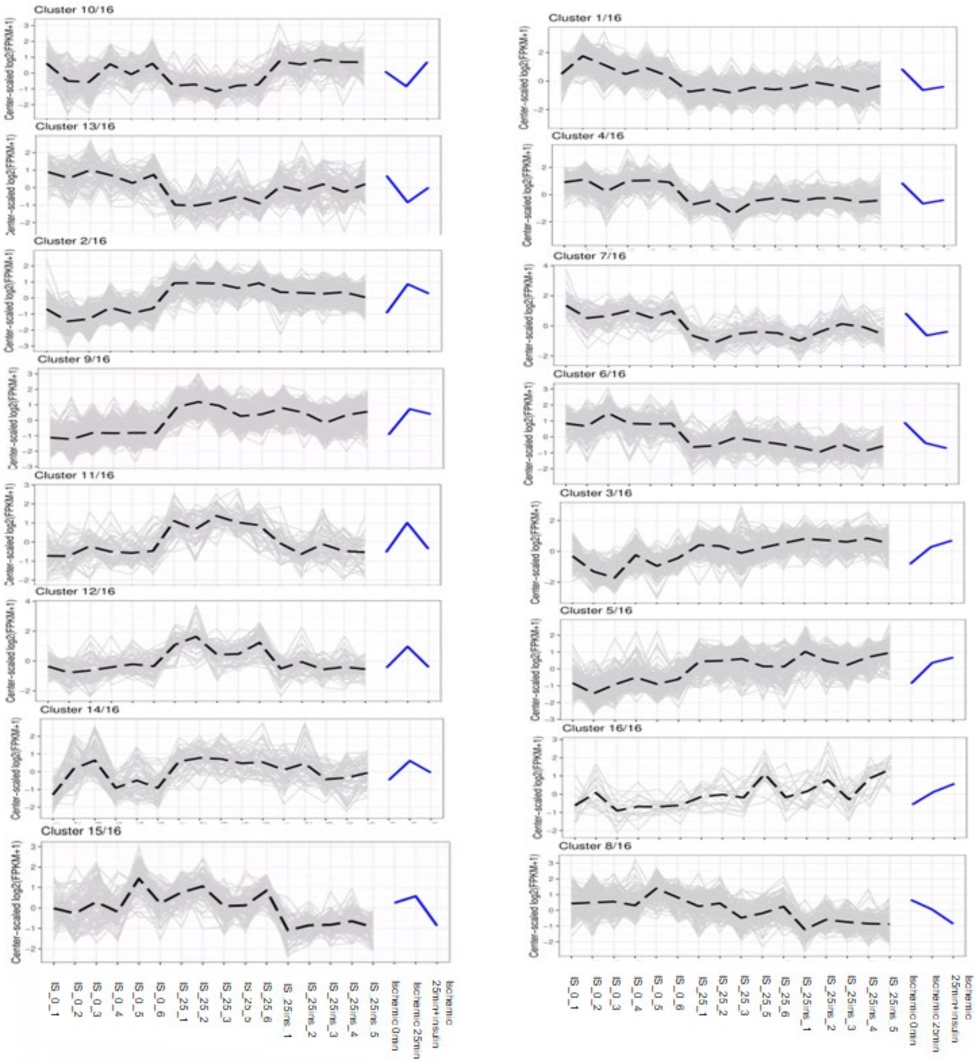

Supplementary Figure 8. Cluster analysis of the categorized differentially expressed genes. n=6 in IS-0 group, n=5 in IS-25 and IS-25ins groups, respectively.

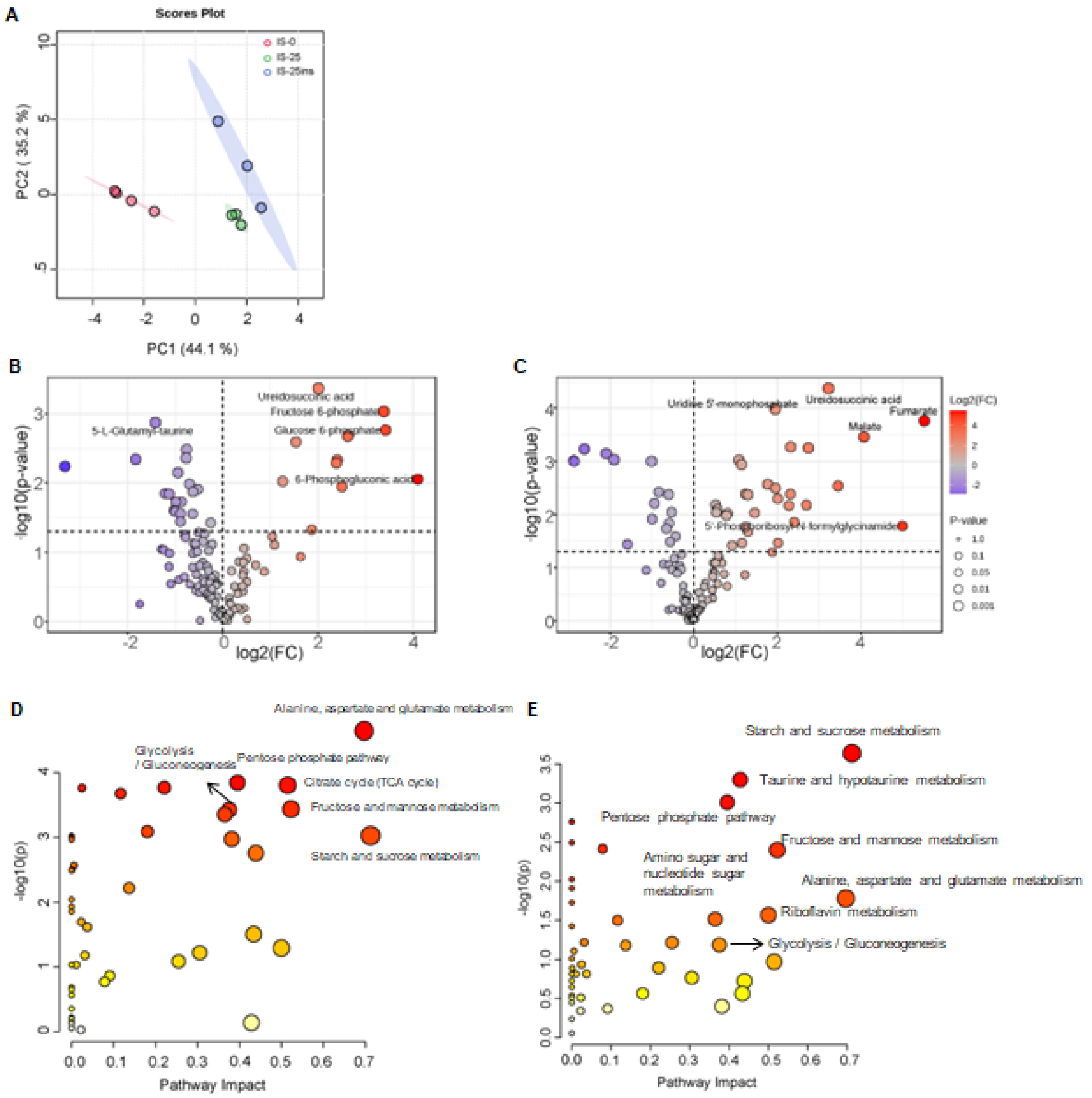

Supplementary Figure 9. Metabolomics analysis of rat right atrial tissue. (A) PCA analysis of data distribution. **(B, C)** Volcano plots for the comparison between IS-0 group and IS-25 group (B), and the comparison between IS-0 group and IS-25 group (C). **(D, E)** Scatter plots of the pathway analysis in the comparison between IS-0 group and IS-25 group (D), and the comparison between IS-0 group and IS-25 group (E). n=4 in IS-0 group, n=3 in IS-25 and IS-25ins groups, respectively.

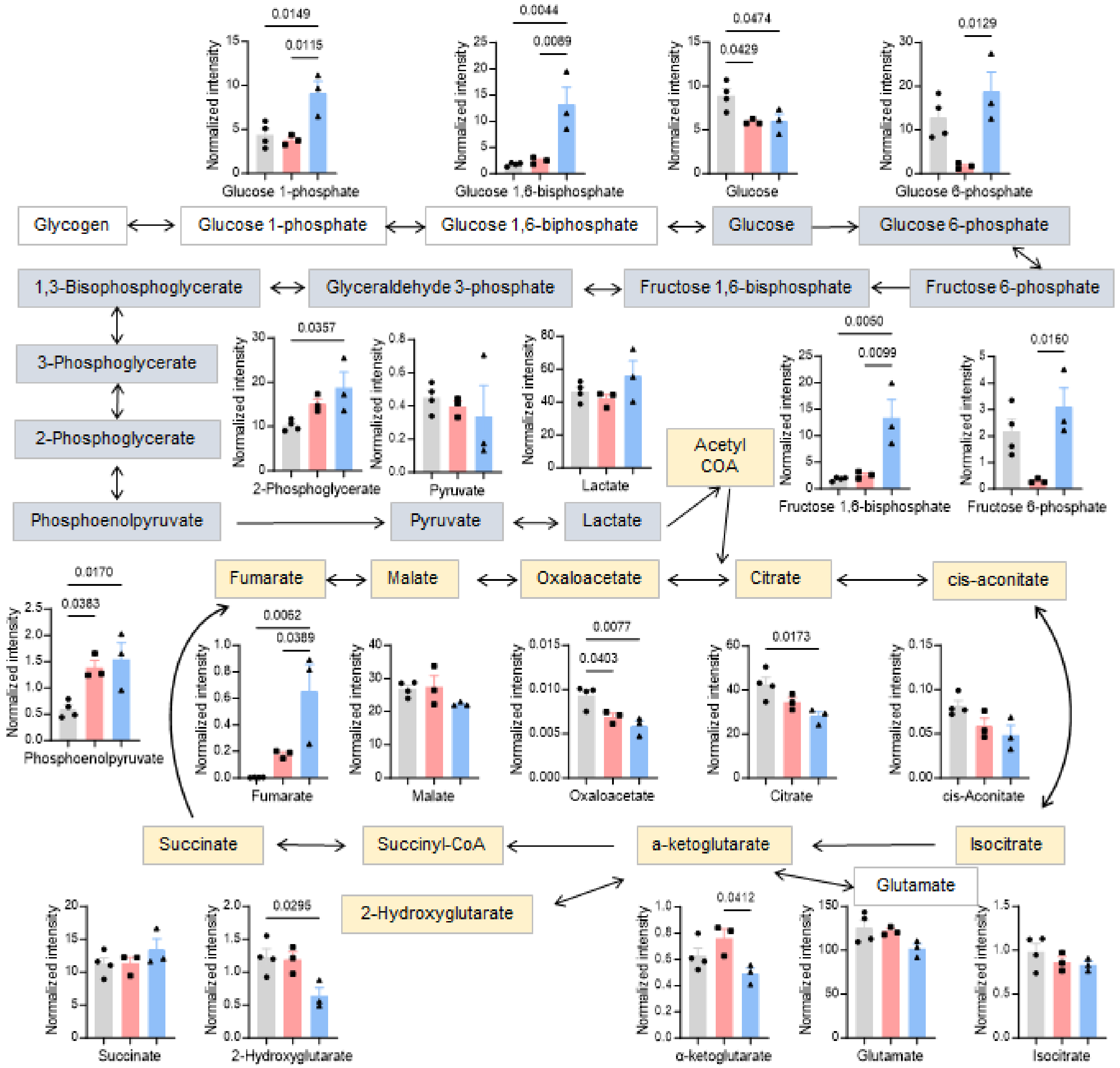

Supplementary Figure 10. Analysis of the intensity of intermediate metabolites in glucose metabolism. The normalized intensity is calculated as the peak area (raw relative abundance) of each metabolite divided by the total peak area of the respective sample, multiplied by 1000. n=4 in IS-0 group, n=3 in IS-25 and IS-25ins groups, respectively. One-way ANOVA with Tukey's multiple comparison was performed.

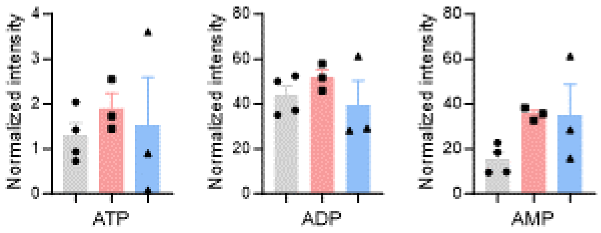

Supplementary Figure 11. Analysis of the abundance of metabolites during energy production in the right atria. n=4 in IS-0 group, n=3 in IS-25 and IS-25ins groups, respectively. One-way ANOVA with Tukey's multiple comparison was performed.

Supplementary table 1. AF inducibility in individual animals as shown in Fig. 1B

	LA (positive rate in technical repeats)						RA (positive rate in technical repeats)					
Rat#	1	2	3	4	5	6	1	2	3	4	5	6
IS-0	1/5	0/5	0/5	0/5	0/5	0/5	0/5	0/5	0/5	0/5	2/10	2/10
IS-10	0/5	0/5	0/5	0/5	1/5	0/5	1/5	1/5	0/5	0/5	0/5	1/5
IS-25	1/5	1/5	0/5	1/5	0/5	0/5	3/5	4/5	4/5	5/5	5/5	3/5

Supplementary table 2. AF inducibility in individual animals as shown in Fig. 2B

	RA (positive rate in technical repeats)					
Rat#	1	2	3	4	5	6
IS-25	5/5	5/5	4/5	3/5	4/5	1/5
IS-25ins	1/5	0/5	0/5	0/5	1/5	1/5
11.2GLU	4/5	5/5	4/5	4/5	1/5	2/5
PRV	1/5	2/5	4/5	4/5	4/5	3/5
PRVins	1/5	1/5	0/5	0/5	0/5	1/5
Insulin$^{-reperfusion}$	1/5	5/5	4/5	1/5	4/5	4/5
